# Differences in levels of phosphatidylinositols in healthy and stable Coronary Artery Disease subjects revealed by HILIC-MRM method with SERRF normalization

**DOI:** 10.1371/journal.pone.0252426

**Published:** 2021-06-04

**Authors:** Yue Huang, Ruipeng Mu, David Wen, Joseph S. Grimsby, Meina Liang, Anton I. Rosenbaum

**Affiliations:** 1 Integrated Bioanalysis, Clinical Pharmacology and Quantitative Pharmacology, Clinical Pharmacology & Safety Sciences, R&D, AstraZeneca, South San Francisco, CA, United States of America; 2 Research and Early Development, Cardiovascular, Renal and Metabolism (CVRM), BioPharmaceuticals R&D, AstraZeneca, Gaithersburg, MD, United States of America; Pacific Northwest National Laboratory, UNITED STATES

## Abstract

Quantification of endogenous biomarkers in clinical studies requires careful evaluation of a number of assay performance parameters. Comparisons of absolute values from several clinical studies can enable retrospective analyses further elucidating the biology of a given biomarker across various study populations. We characterized the performance of a highly multiplex bioanalytical method for quantification of phosphatidylinositols (PI). Hydrophilic interaction chromatography (HILIC) and multiple reaction monitoring (MRM) were employed for targeted multiplex quantification. Odd-chain PI species that are not normally present in human plasma were utilized as surrogate analytes (SA) to assess various assay performance parameters and establish a definitive dynamic linear range for PI lipids. To correct for batch effects, Systematic Error Removal using Random Forest (SERRF) normalization algorithm was employed and used to bridge raw values between two clinical studies, enabling quantitative comparison of their absolute values. A high throughput method was developed, qualified, transferred to an automation platform and applied to sample testing in two clinical trials in healthy volunteers (NCT03001297) and stable Coronary Artery Disease (CAD, NCT03351738) subjects. The method demonstrated acceptable precision and accuracy (±30%) over linear range of 1–1000 nM for SA and 8-fold dilutional linearity for endogenous PI. We determined that mean-adjusted average QC performed best for normalization using SERRF. The comparison of two studies revealed that healthy subject levels of PI are consistently higher across PI species compared to CAD subjects identifying a potential lipid biomarker to be explored in future studies.

## Introduction

Lipids are a major component of plasma [1]. The concentration and profile of plasma lipids are related to the individual’s diet [2] and system metabolism that reflects multiple aspects of the individual’s health status [3, 4]. Plasma phospholipids may function as biomarkers for a variety of diseases, such as non-alcoholic fatty liver disease [5], type 2 diabetes [6, 7], cardiovascular diseases [2, 8], certain cancer types [9, 10], Alzheimer’s disease [11–13], depression [14] and autism [15]. Phosphatidylinositols (PI) represent a small percentage of plasma phospholipids [1]. Nonetheless, PI is the biosynthetic precursor for glycosyl-phosphatidylinositol (GPI), which serve as anchors for proteins and important signaling lipids–phosphatidylinositides (PIP) [16]. The biosynthesis and metabolism of PI and its derivatives have been extensively studied [17]. Recent research showed that high density lipoprotein (HDL) particles contain approximately 0.5–1.5% (wt.) of PI [18]. As a negatively charged phospholipid, PI may significantly impact the charge-dependent interactions of HDL with lipases and other proteins and oral administration has shown to increase HDL-C in normolipidemic subjects [19]. Although the signaling role of PI itself is unclear, PI remains a potentially important biomarker of clinical interest. Therefore, careful characterization of the quantitative properties of a high throughput bioanalytical method for the measurement of human plasma PI is valuable. Technical innovation has advanced a variety of methods for lipid quantification [20–24]. Among these, mass spectrometry (MS)-based approaches have been deployed following two main strategies: chromatography-based separation or direct infusion, often in conjunction with ion-mobility spectrometry (IMS) separation. Direct infusion has the benefits of fast sample analysis and high throughput [25, 26]. The chromatographic separation methods usually have lower throughput but may provide additional specificity and selectivity. The common chromatographic separation methods used for quantitative lipid analysis include normal phase liquid chromatography (NPLC) [22], reverse phase liquid chromatography (RPLC) [27] and hydrophilic interaction liquid chromatography (HILIC) [28–30] as well as multi-dimensional separation [31, 32]. Each method has its unique separation mechanisms and applications.

Herein, we present comprehensive quantitative analytical characterization of a high throughput multiplex method with HILIC separation coupled with multiple reaction monitoring (MRM) in negative mode for measurement of major PI species in human plasma. Additionally, this method features a novel automation-compatible isopropanol (IPA) extraction procedure. A total of 31 endogenous PI species were monitored. To better characterize the dynamic range of the assay we employed both surrogate analytes (SA) and endogenous PI species. This dual approach was employed since the endogenous levels of PI species span a wide range of concentrations, and it is not practical to formally examine method selectivity and accuracy for each PI species, due to the lack of authentic reference standards and high endogenous concentrations in plasma. The SA approach enables true evaluation of response-concentration for SA over a wide concentration range (1–1000 nM). The evaluation using endogenous PI species confirmed that the method supported linear quantification range over 8-fold dilution across 30 different PI species. Method extraction recovery and the stability of the samples were also evaluated. Furthermore, the method was automated for large-scale clinical sample analysis. Patient samples from two clinical studies [33–35] were analyzed with this method. In order to correct for batch effects observed during clinical sample analysis we examined the performance of the recently published Systematic Error Removal using Random Forest (SERRF) algorithm [36] for normalization of batch-to-batch variability across the two studies.

## Material and methods

The studies (Clinicaltrials.gov identifier: NCT03001297, NCT03351738) were performed in accordance with ethical principles that have their origin in the Declaration of Helsinki and are consistent with International Council for Harmonization (ICH)/Good Clinical Practice (GCP), and applicable regulatory requirements, and have been approved by the ethics committees/institutional review boards of the participating centers. All participants provided written informed consent. The pooled human plasma and individual healthy human plasma were purchased from BioIVT.

Samples were extracted using IPA-containing internal standard (IS) PI (12:0|13:0) and analyzed using Nexera X2 UHPLC system (Shimadzu, Kyoto, Japan) with 6500+ QTRAP mass spectrometer (SCIEX, Framingham, MA). Chromatographic separation was performed on an Acquity UPLC BEH HILIC Column (130 Å, 1.7 μm, 2.1 mm X 100 mm, Waters, Milford, MA). SERRF has been implemented largely as described [36] with some modifications. Automation was implemented using Bravo Liquid Handling Platform (Agilent).

Detailed Information on Methods, Reagents, Instrumentation and Software can be found in S1 File.

## Results

A standard curve defines the mathematical relationship between analyte concentration and assay response and thus enables absolute quantification of an analyte. However, absolute multiplex quantification for lipids is challenging due to the isomeric complexity of the individual lipid species, lack of authentic well-characterized standards and high levels of endogenous lipid species. Although quantification via normalization to the IS concentration/amount cannot provide truly absolute concentration quantification, it can provide useful comparative information when the linearity range of the method, the stability of the target lipid analyte, assay reproducibility and the impact of batch effects have been carefully examined.

There are several practical difficulties for the qualification of a bioanalytical method for lipids as clinical biomarkers. One major concern is the variability of different endogenous lipid concentration affecting the accurate quantification for low concentration lipids. Despite the high levels of total lipids in plasma, there are large concentration differences between individual lipid classes and species within each class. If the targeted lipid analyte is present at relatively low concentrations, other co-existing plasma lipids can contribute to background variability. With lipidomic approaches aiming at full lipid profiling, proper quantitative characterization of lower abundance lipids for use as clinical biomarkers is an area of emerging interest that requires further inquiry. The other challenge, which is common for large-scale studies, is the requirement for quantification reproducibility over the long term of the clinical study. Herein, we addressed these concerns by refining extraction procedures, qualifying the method’s dynamic range and introducing multiple layers of normalization.

### Development of the extraction method

Folch method [37] is a classic extraction procedure employed for the analysis of various lipids, including PI. The method, although recognized as one of the most comprehensive extraction methods for all lipid classes, requires the use of volatile, hazardous liquids and a sophisticated preparation procedure. One of the modified procedures, BUME, relies on butanol/methanol/heptane solvent system with acetic acid [38]. Recently, MTBE extraction method, using methyl tert-butyl ether/methanol/water was also reported [37]. These methods simplified the extraction process with the aim to reduce experimental variances. Since PI lipids are relatively polar, we evaluated extraction of PI species from plasma using IPA and compared the results with both BUME and MTBE methods. IPA extraction was proven to generate similar signal intensity on the major PI species as the BUME extraction method or the MTBE method (S1 Fig in S1 File). This extraction method employing IPA provided robust extraction procedure and reproducible experimental results while using a less toxic and less volatile liquid. To support high throughput sample analysis required for large clinical studies, this method was transferred to an automation platform (e.g. Agilent BRAVO).

In order to select the internal standards (IS) and surrogate analytes (SA), several synthetic PI species containing odd-chain acyl chains or isotopic labels were tested for selectivity, specificity and linearity of response over a concentration range. A total of five PI species, PI (12:0|13:0), PI (17:0|14:1), PI (21:0|22:6), PI (17:0|20:4) and PI (16:0|18:1)-D31 were tested with and without plasma extract. Based on the signal intensity and selectivity from endogenous species, PI (12:0|13:0) had least interference with endogenous PIs and was selected as IS, while PI (17:0|14:1) and PI (21:0|22:6) were selected as SA. While SA possess chemical and physical properties similar to the endogenous PI species, no interference from the human plasma extract was detected (S2 Fig in S1 File). To select the relevant PI species that are commonly seen in human plasma, MRM transitions were first predicted using Lipidview™ software for PI with total chain length 30–40 and total unsaturation up to 6 (generating list of “MRM list-All”). A pooled lot of human plasma was analyzed with “MRM list-All”, and 31 endogenous PI species with highest signal intensity at were monitored (S1 Table in S1 File).

### Method linear dynamic range assessment with SA and endogenous PI

To quantitatively compare changes in PI concentrations a linear range of the method must be established. Since there will be multiple PI species monitored in this experiment and the concentration of these endogenous compounds can vary, SA serial dilution was used to first characterize the entire linear dynamic range of the method. Two SAs were spiked into reconstitution solution and extracted blank plasma at nine concentration levels between 0.5–1000 nM (0.5, 1, 4, 10, 40, 100, 250, 500, 1000 nM) in the presence of IS. Analyte/IS PAR were used to evaluate the linear dynamic range across the concentration range. Since during sample analysis PI concentration changes would be compared longitudinally without an external calibration curve, weighing should not be applied.

**Fig 1:**
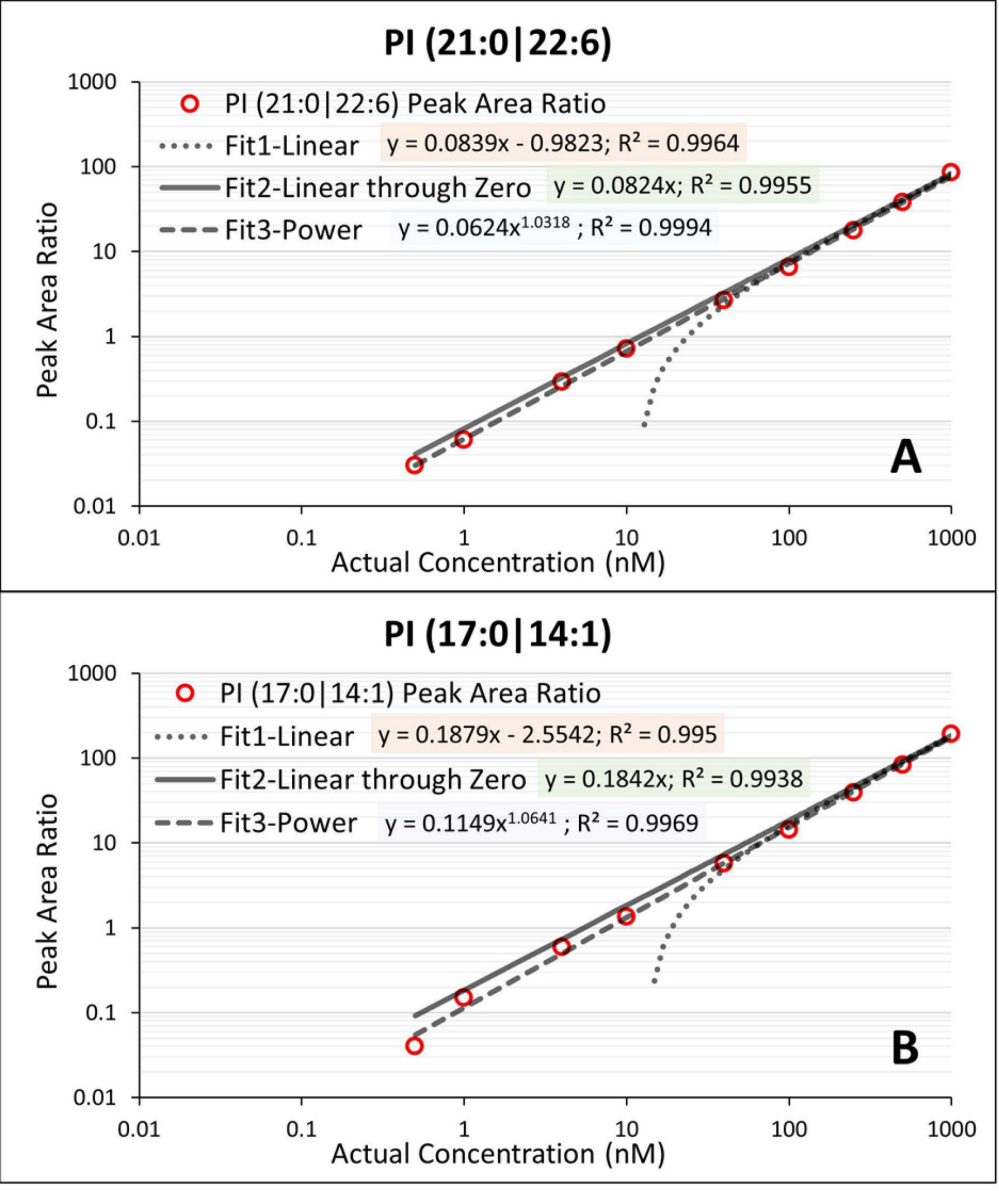
Linearity evaluation with surrogate analytes A) PI (21:0|22:6) and B) PI (17:0|14:1).

Three different linear fits were evaluated with the SA dataset (Fig 1). The recovery results for each of the standard curves (S3 Table in S1 File) were used to evaluate the linearity of the method. The simplest linear fit (i.e., fit 1) exhibited significant bias at the lower concentration range despite having an acceptable correlation coefficient value (R^2^) because no weighing was applied. However, if the y-intercept is forced to zero, the linear-through-zero fit (i.e., fit 2) generated reasonable recovery and R^2^-value for both SA. Among the three fitting algorithms evaluated, the power (or linear log(y)-log(x) fit, i.e., fit 3) generated the best R^2^-value and recovery results. However, when evaluated with endogenous PI species using human plasma dilution samples, the fit 2 and 3 generated comparable accuracy. Therefore, the linear through zero fit appropriately describes the applicable data analysis when comparing fold changes between study samples. These data demonstrate that for PI species, the absolute signal responses quantified as peak area ranging from mid-10^3^ to mid-10^6^ (cps×s) are within the linear dynamic range of our method. The result from this experiment is used as guidance in designing the extraction and dilution of endogenous PI species. Based on this experiment, the extraction procedure was finalized to allow the method response of the target endogenous PI species to fall in the linear dynamic range. After establishing the linear dynamic range of the method with SA, the linearity of response for the endogenous PI species was evaluated. Parallel dilution of human pooled plasma was performed at 6 levels: 8, 6, 4, 3 and 2-fold dilutions, as well as the undiluted human plasma serving as the highest concentration of endogenous PI species with PBS containing 40 mg/mL BSA serving as diluent.

**Fig 2:**
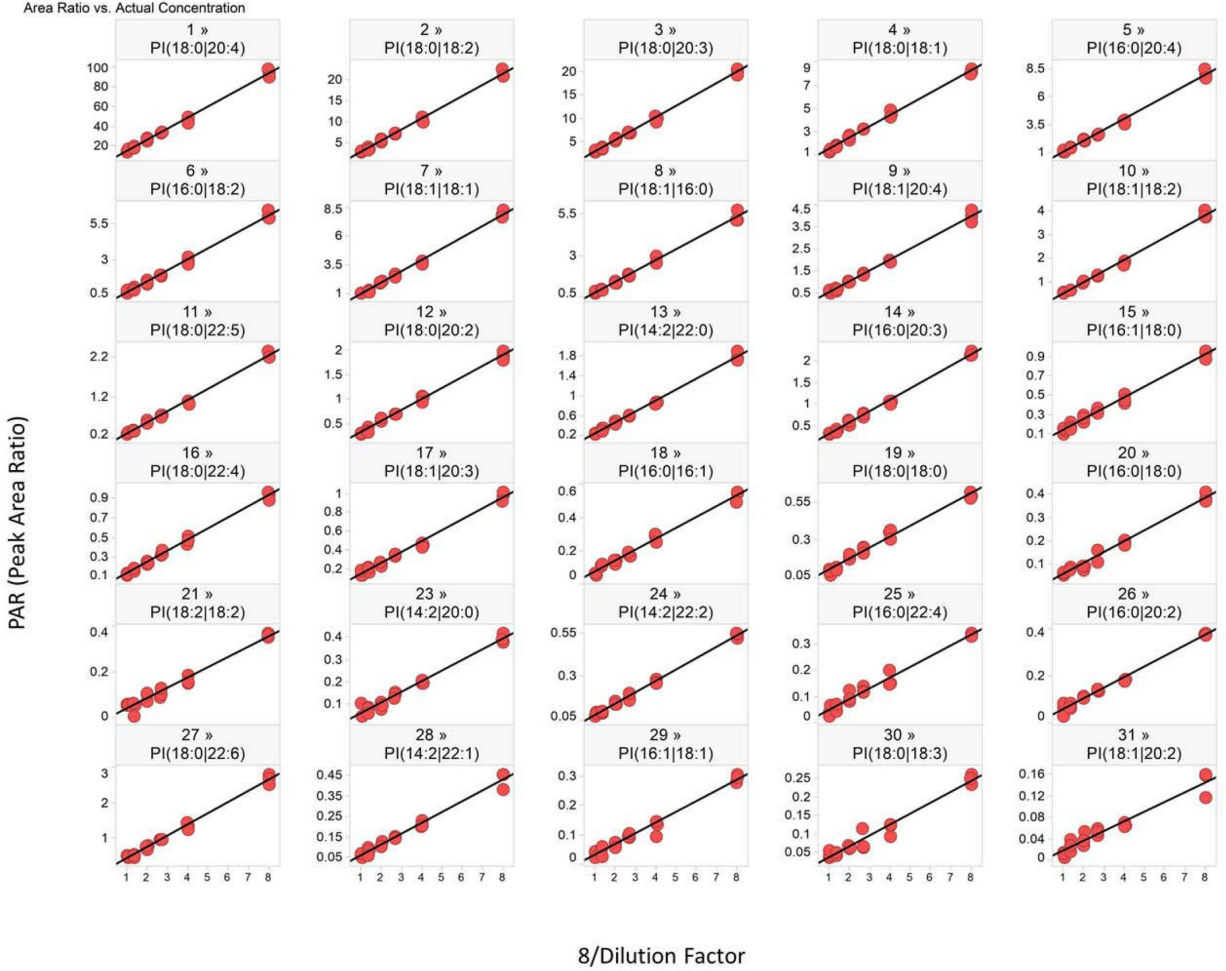
Linearity evaluation with parallel dilution series (1x, 2x, 3x, 4x, 6x, 8x) of endogenous human plasma PIs. The linear regression of 30 PI species were monitored, with exception of PI (16:0|16:0) which was omitted from analysis due to poor response.

The linearity of response for the endogenous PI species is presented in Fig 2. Among 31 endogenous PI species, 30 species showed a linear relationship over 8-fold dilution range (except PI 16:0|16:0). Since the clinical data would be expressed as fold-change from baseline over time for each subject, the fold-change for each individual sample as the result of dilution was also evaluated. For each PI lipid monitored, the fold-change between the PAR from two samples are compared against the nominal fold-change. The result demonstrates the accuracy of fold-change calculation. The data for each pair of samples are summarized in S4 Table and S3 Fig in S1 File. The total number of values outside of 70–130% accuracy was 10% of the entire dataset. From the results, the majority (>70%) of the monitored PI species showed a 10^th^ and 90^th^ percentile of the fold change recovery value (P10 and P90, respectively) in between 0.7–1.3. From the statistical analysis of the data set for each individual lipid species, 28 out of 31 showed a 95% confidence interval within the range of 0.8–1.2. Generally, highly accurate fold-change measurements for more abundant species were observed, while less accurate recovery was observed for the lipid species with generally lower signal intensity. To establish a robust method for large-scale clinical sample testing, evaluation of analyte stability under various conditions relevant to sample analysis is important. Therefore, plasma sample stability and extracted sample stability in autosampler were evaluated (S4 Fig in S1 File).

**Fig 3:**
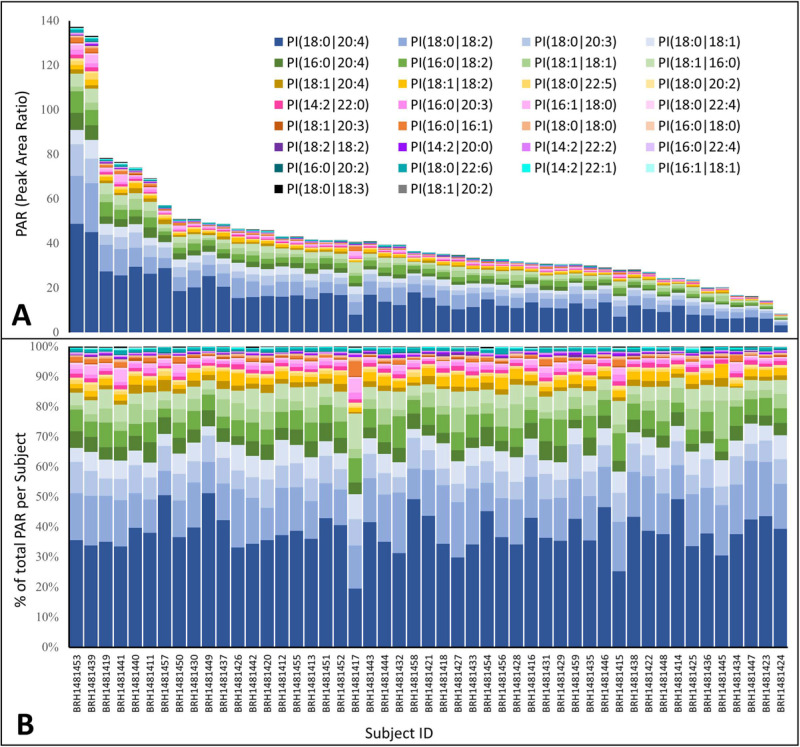
PAR distribution of 30 endogenous PI lipids for 49 individual human plasma. A) Stacked PAR of each PI species. B) Percentage of each PI species calculated from normalization to the total PAR of each individual.

A set of 49 individual human plasma samples with various HDL and LDL levels were analyzed using the method described herein to characterize the distribution of the PI species in the healthy population (S5 Table in S1 File and Fig 3). The detailed demographic, general lipid data and reference range for each PI species for the healthy donors can be found in the S6 and S7 Tables in S1 File. More than 10-fold differences in concentration of PI species between different individuals have been observed (Fig 3A) leading to broad reference ranges for PI species. Therefore, a longitudinal study of the PI lipid profile change for each individual may provide more clinically-relevant information. Thus, for interventional studies it is important to acquire the baseline sample for each patient. Nonetheless, the distribution itself of the PI species seems less variable among the healthy subjects. When normalized to the total PI concentration of all species, the distribution of each PI species is reproducible between individuals (Fig 3B).

### Application to clinical studies

The methods were also applied to the measurement of PI species in two clinical trials: NCT03001297 (SAD) [33] and NCT03351738 (MAD) [34]. For clinical sample analysis, all samples were diluted 8x with PBS-40 mg/mL BSA. One lot of pooled human plasma was used to generate QC samples for both studies. Pairs of LQC and HQC were spread among the test samples to monitor experimental quality. The sequence of injections was designed as follows: 1) system suitability samples, 2) first set of LQC and HQC, 3) samples and one set of LQC and HQC every 15 samples, 4) final set of LQC and HQC, 5) system suitability injection. The acceptable range for the ratio of HQC PAR to LQC PAR for each species was within 70–130% of the expected 8-fold change. The levels of PI quality controls (LQC and HQC) relative to the study samples can be found in S5 Fig in S1 File. A majority of samples analyzed for the majority of endogenous PI species were within the range of the method defined by the HQC and LQC. Some variability between the batches was observed, especially for batches acquired during sample analysis of the two clinical studies, which were analyzed at two different testing sites within AstraZeneca.

**Fig 4:**
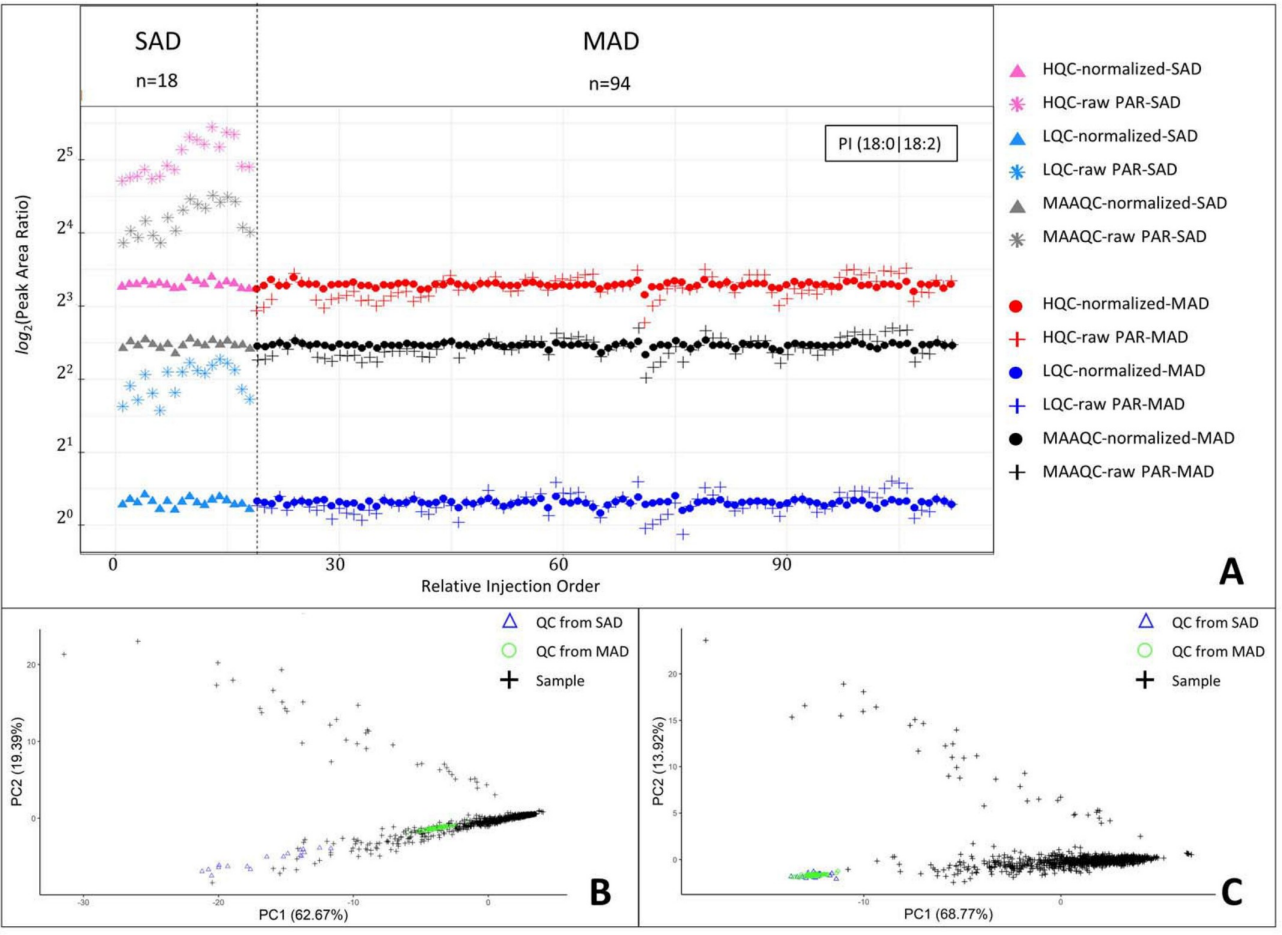
A) Normalized PAR from SAD and MAD studies can be bridged with SERRF normalization with LQC, HQC or MAAQC. B) PCA analysis for QCs and samples before SERRF normalization from SAD and MAD studies. C) PCA analysis for QCs and samples after SERRF normalization from SAD and MAD studies.

**Fig 5:**
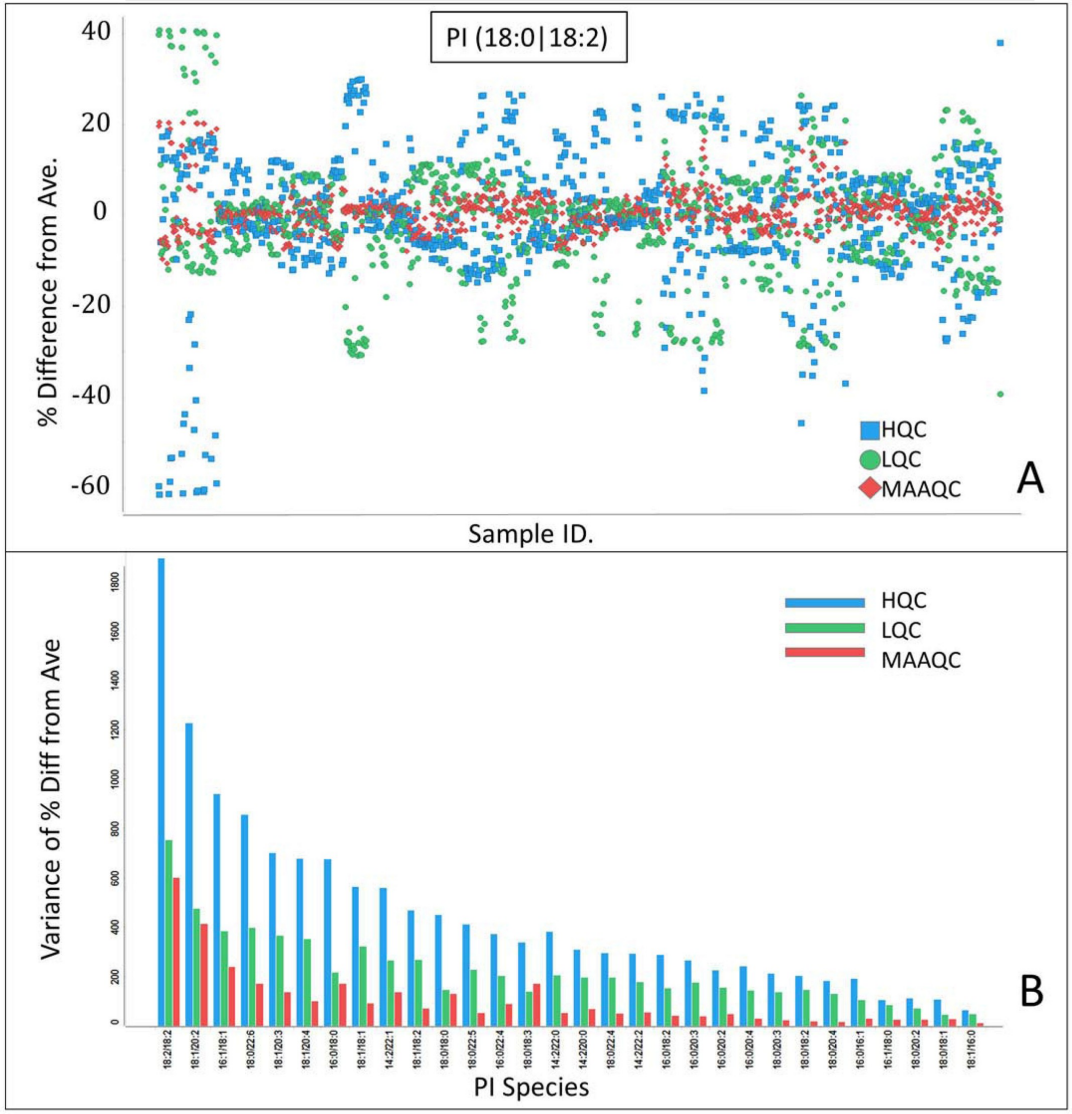
A) Percent Difference of each normalized value using LQC, HQC and MAAQC from the average of these three values for MAD study samples, using PI (18:0|18:2) as an example. B) The variance of % difference from average for each PI species.

In order to correct for the batch-to-batch variability and explore the possibility of bridging the raw data between the two clinical studies, we evaluated the recently published SERRF normalization algorithm [36]. First, we compared SERRF normalization to other methods using LQC, HQC as well as MAAQC for the SAD study. SERRF outperformed the other normalization methods evaluated for this dataset (S6 Fig in S1 File). This preliminary evaluation indicated that either level of QC enabled normalization by SERRF. The normalization improvement was also evident in the changes in QCs after correction using SERRF in both SAD and MAD studies (S7 Fig in S1 File). This was consistent across PI species (S8A Fig in S1 File). Moreover, SERRF-based normalization maintained the HQC/LQC intensity ratio of 8 well (S8B Fig in S1 File). This further supports that we indeed have established a linear dynamic range of the assay within the 8x range of the LQC-HQC samples. Additionally, we have endeavored to bridge the results from the two clinical studies. As shown in Fig 4, SERRF successfully bridged data from two different studies enabling direct comparison of the raw data (rather than merely fold change) between the two studies. Interestingly, upon examining the %RSE from each study (S9 Fig in S1 File), we observed that the variability significantly decreased between SAD and MAD studies as the result of introduction of automation for the analysis of the MAD study. Finally, we examined how SERRF-based correction impacted sample data from the MAD study using either LQC, HQC or mean-adjusted average QC. In order to evaluate SERRF’s impact, we calculated an average value upon normalization using each QC level of each sample for each PI species. Then, the % difference from that average was calculated for each normalization that used a different QC. The % difference data for PI (18:0|18:2) are presented in Fig 5A, and the data for all PI species is presented in S10 Fig in S1 File. In order to further condense the data and enable quantitative comparison of the 3 normalization approaches we calculated the variance of % difference from average which is presented in Fig 5B. From these data it is apparent that mean-adjusted average QC outperforms the normalization by either LQC or HQC when applied to actual study samples across PI species. It is noteworthy that LQC also outperformed HQC in this evaluation, a likely consequence of the fact that LQC was generally closer to study samples for this study (S5 Fig in S1 File). Furthermore, to ensure that the SERRF normalization enabled accurate comparison of these two datasets we compared the data for each PI species for the set of 49 individual human plasma samples (S7 Table in S1 File) that were run along the samples from the two clinical studies. The % difference between the two datasets was calculated as follows for each PI species for each sample:

$$\%Difference=100*\frac{{PAR}_{MAD}-{PAR}_{SAD}}{({PAR}_{MAD}+{PAR}_{SAD})/2}$$

Since assay variability was deemed acceptable at 30%, we applied ±35% acceptance criteria for % difference calculated above. As is shown in S11 Fig in S1 File, 12 PI species had 33% or less of samples within these acceptance criteria.

**Fig 6:**
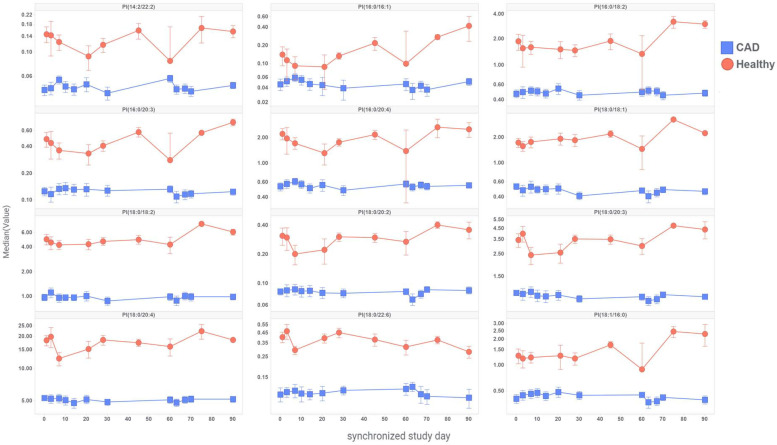
Longitudinal comparison of PI levels between the placebo groups from the two clinical studies after normalization with SERRF. Data are presented as median of PAR (value), error bars are standard error of the mean.

The distribution profile comparison between healthy and CAD population is presented in S12 Fig in S1 File for PI species deemed acceptable from the analysis presented in S11 Fig in S1 File. PI levels were markedly higher in healthy subjects compared to CAD patients across PI species. The difference was determined to be statistically significant by 2-tailed heteroscedastic t-test (p<0.0005 for all 12 PI species presented in S12 Fig in S1 File). Additionally, we evaluated the longitudinal changes in PI species from placebo arms of both studies (Fig 6). These data revealed that PI levels remained relatively stable over period of several months in both patient populations while the lower levels in CAD patients were maintained relative to healthy volunteers in the 12 PI species examined.

## Discussion

In our endeavor to fully characterize the bioanalytical method for the quantification of PI phospholipids for application to clinical bioanalysis, we optimized extraction methods, implemented automation, introduced multiple layers of experiment controls and adopted the SERRF algorithm for data normalization. The extraction method was optimized based on the physicochemical properties of PI to simplify the procedure and to improve reproducibility. The extraction procedure employed a less toxic and volatile solvent (IPA). The method was evaluated with comparison to other lipid extraction methods, followed by assessments of method linearity with both SA and endogenous PI, as well as extraction recovery at several concentrations. This work for the first time comprehensively evaluated key questions of linearity and of method response in the context of PI lipid quantification. Sample stability, run length and re-injection reproducibility were established to guide clinical sample analysis. The reference range for PI species in healthy population was established. The method was transferred to an automation platform to further solidify its reproducibility. In addition to controlling the assay performance using internal standard, LQC and HQC were run together with samples in a defined injection sequence. The method was used to support exploratory biomarker analysis for two clinical studies. In order to correct for batch effects observed during clinical sample analysis, the SERRF normalization method was applied to the acquired data to reduce random and systematic error and to bridge data acquired across studies. Moreover, we carefully evaluated which level of QC was best suited for such normalization and determined that the MAAQC is most appropriate when evaluated against the study samples themselves. The data were used to evaluate the reference range for PI species in healthy subjects as well as to characterize the distribution of PI species. Finally, our analyses culminated in retrospective comparison of the PI levels from the two clinical trials. We discovered that PI levels were significantly and longitudinally consistently lower in the CAD patients compared to healthy subjects. Importantly, approaches presented within this work exemplify the development, characterization and qualification of analytical methods for the quantification of clinically relevant lipid and metabolic biomarkers using highly multiplex, metabolomic-style methods not amenable to full validation as prescribed by regulatory guidance [39, 40]. Further characterization of the underlying biological mechanisms responsible for the decrease of the PI biomarker in CAD patient population relative to healthy subjects as well as in conjunction with pharmacological intervention may reveal more information on this clinically-relevant biomarker.

## Supporting information

S1 File (DOCX)
