# Supplementary material for: Differences in levels of phosphatidylinositols in healthy and stable Coronary Artery Disease subjects revealed by HILIC-MRM method with SERRF normalization
Source: PLoS One. 2021 Jun 4;16(6):e0252426. doi: 10.1371/journal.pone.0252426 (PMC8177664; doi:10.1371/journal.pone.0252426)
Supplement: S1 File — (DOCX) [file pone.0252426.s001.docx]

**S1 File (Supplemental Figures, Tables, Methods) for**

Differences in Levels of Phosphatidylinositols in Healthy and Stable Coronary Artery Disease Subjects Revealed by HILIC-MRM Method with SERRF Normalization

**Supplemental Methods**

**Reagents**

Lipid standards PI(12:0|13:0), PI(17:0|14:1), PI(21:0|22:6), PI(17:0|20:4) and PI(16:0|18:1)-D31 were purchased from Avanti Polar Lipids (Alabaster, AL). HPLC grade water was purchased from JT Baker (Phillipsburg, NJ). HPLC grade isopropanol (IPA), acetonitrile (ACN), butanol, methanol, dichloromethane (DCM), heptane, ethyl acetate (EtAc), methyl-tertbutyl-ether (MTBE), bovine serum albumin (BSA), ammonium acetate, acetic acid and ammonia were purchased from Sigma-Aldrich (St. Louis, MO). Special glass-coated 96-well extraction plates and glass vials with PTFE lining were from Thermo-Fisher (Waltham, MA). Human plasma (pooled and individual) were purchased from BioIVT (Westbury, NY).

**Instrumentation**

The LC-MS/MS system used was Nexera X2 UHPLC system (Shimadzu, Kyoto, Japan) with 6500+ QTRAP mass spectrometer (SCIEX, Framingham, MA). Chromatographic separation was performed on an Acquity UPLC BEH HILIC column (130 Å, 1.7 μm, 2.1 mm X 100 mm, Waters, Milford, MA). The mobile phases used were: MPA (5% water, 95% ACN, v/v) and MPB (50% water, 50% ACN, v/v), both with 10 mM ammonium acetate, pH 8.0-8.5. The separation was performed at 37°C at a flow rate of 0.5 mL/min. The separation gradient was 5% to 13% MPA over 4 min, followed by a 2 min wash period and a 4 min equilibration period.

**Procedures**

MS Parameter Optimization

Synthetic reference standard PI species PI(17:0|14:1) and PI(21:0|22:6) were used to tune the instrument parameters. The highest signal intensity fragment in negative ESI MRM mode monitoring characteristic fragments of acyl chains obtained during the infusion of the synthetic reference standards was matched to their respective chemical structures. Corresponding MRMs for the endogenous PI lipids of interest were predicted using LipidView™ software (SCIEX, Framingham, MA) and are listed S1 Table. After tuning the synthetic reference standards, same MS instrument parameters were used for all PI species (S2 Table).

**Extraction Procedure**

Internal standard (IS) PI (12:0|13:0) was spiked in IPA to make 60 nM IS-IPA solution used as the precipitation solution. One pooled lot of human plasma was used as quality control (QC) samples and prepared at two levels: low QC (LQC, 8x dilution with 40 mg/mL BSA in PBS) and high QC (HQC, undiluted plasma). Test samples were all diluted 8x with 40 mg/mL BSA. 20 μL of LQC, HQC or test sample was precipitated with 180 μL of IS-IPA. Samples were shaken vigorously for about 10 min at 900~1200 rpm. Then, samples were centrifuged for 5 min at 2500 g. After centrifugation, 20 μL of the supernatant was then transferred to 140 μL of reconstitution solution (90.25 % ACN, 9.75 % water, v/v; 10 mM ammonium acetate). Samples were then agitated for 5 minutes at 600 rpm.

The extraction recovery was evaluated at various concentrations with the SA, through the comparison of the peak area ratios (PAR) from pre-extraction and the post-extraction. The extraction recovery compared the PAR of pre-extraction fortified samples, including fortified in plasma and fortified in precipitation solution, to the PAR of post-extraction fortified samples, which represented 100% recovery. The three concentration levels evaluated were approximately 0.88 nM, 8.8 nM and 88 nM in the post-extraction solution. The concentration of the different spike solutions was taken into calculation for the final results. The extraction recovery values assessed by PI (21:0|22:6) were above 80% for the three concentration levels evaluated. For PI (17:0|14:1), the extraction recovery was above 65% across three concentration levels.

Extraction using 3 different methods (IPA, methanol-tert-butyl methyl ether (MTBE) and butanol/methanol (BUME) ) was performed and results presented in S1 Fig.

**MTBE extraction procedure:**

Cyno plasma sample (80 uL) with 10 µL of internal standard solution and 600 µL of methanol. The sample was shaken for 10 min and allowed to sit for 10 min before 2000 µL of MTBE were added to the sample. After shaking, the mixture was allowed to sit under ambient temperature for 1 hour and then vortexed. DI water (500 µL) was added to the mixture to induce phase separation. After 10 min incubation in ambient temperature, the mixture was centrifuged at 1000 *g* for 10 min before approximately 1600 µL of the upper organic phase was collected. The lower water phase was then re-extracted with 800 µL of a MTBE/methanol/water mixture (MTBE/Methanol/Water= 10/3/2.5). Approximately 800 µL of the upper organic phase were collected from the re-extraction. The organic phases collected were combined and dried under nitrogen at 30 ⁰C.

**BUME extraction procedure:**

Cyno plasma sample (80 µL) was mixed with 10 µL of internal standard solution. The following solvents were added to the mixture in order and mixed after each addition: 300 µL (Butanol: Methanol= 3:1), 150 µL (heptane: EtAc= 3: 1), 150 µL (heptane: EtAc= 3: 1), 300 µL (1% acetic acid). The mixture was allowed to sit for 20 min under ambient temperature. After phase separation, 360 µL of the upper organic phase was transferred to a separate tube. To the remaining water phase, re-extraction was performed twice, with 320 µL and 250 µL of heptane and EtAc mixture (heptane: EtAc= 3: 1), respectively. All organic phases collected from the extraction and re-extractions were combined and dried under nitrogen at 30⁰C.

**IPA extraction procedure:**

Cyno plasma sample (80 uL) was mixed with 10 µL of internal standard solution. Then 450 µL of isopropanol was added to the sample and vortexed for 5min. The mixture was centrifuged at 2500 g for 10 min. Then 450 µL of the supernatant was extracted and dried with nitrogen at 30⁰C.

**Data Analysis**

Calculation of Data

Chromatograms were integrated automatically using SCIEX MultiQuant MQ4 algorithm. PAR was calculated for all PI species against the IS. A single lot of pooled human plasma was used for QC samples for both clinical studies.

**Batch-Correction/Normalization using SERRF**

SERRF has been implemented largely as described^35^ with the following modifications. Instead of peak areas, analyte/IS PAR for each PI species were used. Instead of a single level of QC, both LQC and HQC were evaluated for normalization. Additionally, we have evaluated normalization based on a mean-adjusted average QC (MAAQC) calculated as follows, where $\overline{LQC}$ and $\overline{HQC}$ represent the mean LQC and mean HQC of the dataset, respectively.

$MAAQC=\frac{{LQC}/\overline{LQC}+{HQC}/\overline{HQC}}{2}$

**Automation Implementation**

The method was adapted to the Agilent (Santa Clara, CA) Bravo Automated Liquid Handling System with Series III 96 LT Disposable Tip Head (referred as Bravo System) to support clinical study NCT03351738 sample testing. All automation protocols were programmed using VWorks software (version 13.1.1).

Test and HQCs samples were manually loaded into the glass-coated 96-well plates. Bravo System was used to prepare LQC samples by thoroughly mixing 20 µL of pooled human plasma with 140 µL of 40 mg/mL of BSA. Subsequently, all test samples were simultaneously diluted 8-fold with 40 mg/mL BSA. LQC, HQC and test samples (20 µL) were transferred to a separate glass-coated 96-well plate, following the predesignated plate map, with QC samples evenly distributed among test samples. IS-IPA solution (180 µL) was added simultaneously to each well and mixed thoroughly. The plate was then removed from the Bravo System and shaken vigorously for 10 min at 1200 rpm, then centrifuged for 5 min at 2500 *g*. After centrifugation, the plate was returned to the Bravo System. Reconstitution solution (140 µL) was loaded to a third plate. 20 μL of the supernatant from the extraction plate was dispensed to the third plate and mixed with the reconstitution solution. The plate was removed from Bravo system and agitated for 5 minutes at 600 rpm before the LC-MS/MS injection.

A special pipetting technique was employed by the Bravo System when transferring volatile solutions with low viscosity, such as IS-IPA. Before aspiration of the solution, the tips were rinsed three times with an IPA solution to establish a vapor-pressure equilibrium. After aspiration of the reconstitution solution, 20 µL of air gap was aspirated to avoid liquid dripping.


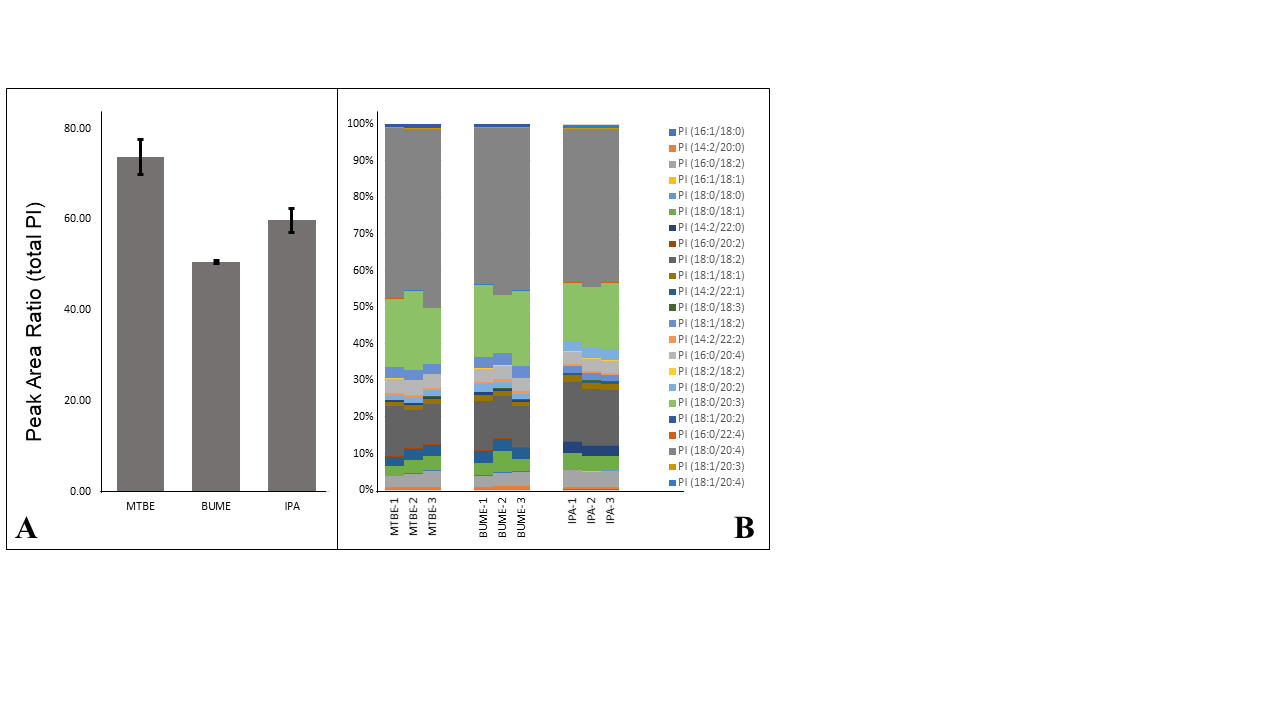


**S1 Fig**. **PI species extracted with three different methods**. A) total PI peak area ratio average and standard deviation (n=3) between MTBE, BUME and IPA extraction. B) Major PI species normalized percentage between three extraction methods, with three replicates for each method.


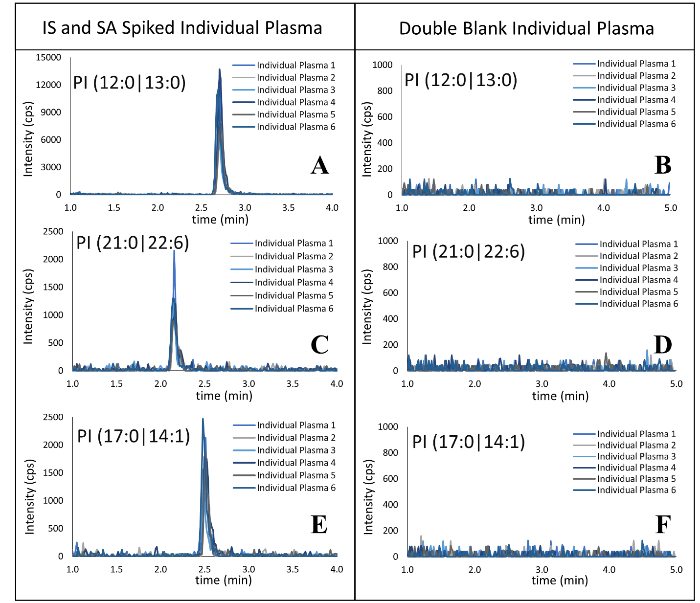


**S2 Fig. Background interference and selectivity evaluation for internal standard PI (12:0|13:0) and surrogate analyte PI (21:0|22:6) and PI (17:0|14:1) in six individual human plasma**. A), C) and E): Internal standard (spiked in precipitation solution at 60 nM) and surrogate analytes (spiked in plasma at 1 nM) in 6 lots of human plasma. B), D) and F): Double blank showed no significant interference for IS and SA.

**S1 Table. List of IS, SA and endogenous PI species of the method**

| Index | PI Species | Analyte Type | Q1/Q3 |
| --- | --- | --- | --- |
| IS | PI (12:0\|13:0) | internal standard | 711.4 / 213.2 |
| SA1 | PI (17:0\|14:1) | surrogate | 793.5 / 269.2 |
| SA2 | PI (21:0\|22:6) | surrogate | 951.6 / 325.3 |
| 1 | PI (18:0\|20:4) | endogenous | 885.6 / 283.3 |
| 2 | PI (18:0\|18:2) | endogenous | 861.6 / 279.2 |
| 3 | PI (18:0\|20:3) | endogenous | 887.6 / 283.3 |
| 4 | PI (18:0\|18:1) | endogenous | 863.6 / 283.3 |
| 5 | PI (16:0\|20:4) | endogenous | 857.5 / 255.2 |
| 6 | PI (16:0\|18:2) | endogenous | 833.5 / 255.2 |
| 7 | PI (18:1\|18:1) | endogenous | 861.6 / 281.2 |
| 8 | PI (18:1\|16:0) | endogenous | 835.5 / 255.2 |
| 9 | PI (18:1\|20:4) | endogenous | 883.5 / 281.2 |
| 10 | PI (18:1\|18:2) | endogenous | 859.5 / 281.2 |
| 11 | PI (18:0\|22:5) | endogenous | 911.6 / 283.3 |
| 12 | PI (18:0\|20:2) | endogenous | 889.6 / 283.3 |
| 13 | PI (14:2\|22:0) | endogenous | 861.6 / 223.2 |
| 14 | PI (16:0\|20:3) | endogenous | 859.5 / 255.2 |
| 15 | PI (16:1\|18:0) | endogenous | 835.5 / 283.3 |
| 16 | PI (18:0\|22:4) | endogenous | 913.6 / 283.3 |
| 17 | PI (18:1\|20:3) | endogenous | 885.6 / 281.2 |
| 18 | PI (16:0\|16:1) | endogenous | 807.5 / 255.2 |
| 19 | PI (18:0\|18:0) | endogenous | 865.6 / 283.3 |
| 20 | PI (16:0\|18:0) | endogenous | 837.6 / 255.2 |
| 21 | PI (18:2\|18:2) | endogenous | 857.5 / 279.2 |
| 22 | PI (16:0\|16:0) | endogenous | 809.5 / 255.2 |
| 23 | PI (14:2\|20:0) | endogenous | 833.5 / 223.2 |
| 24 | PI (14:2\|22:2) | endogenous | 857.5 / 223.2 |
| 25 | PI (16:0\|22:4) | endogenous | 885.6 / 255.2 |
| 26 | PI (16:0\|20:2) | endogenous | 861.6 / 255.2 |
| 27 | PI (18:0\|22:6) | endogenous | 909.6 / 283.3 |
| 28 | PI (14:2\|22:1) | endogenous | 859.5 / 223.2 |
| 29 | PI (16:1\|18:1) | endogenous | 833.5 / 281.2 |
| 30 | PI (18:0\|18:3) | endogenous | 859.5 / 283.3 |
| 31 | PI (18:1\|20:2) | endogenous | - 1. / 281.2 |

**S2 Table. MS Instrument Parameters**

| Instrument parameter | Value |
| --- | --- |
| Ionization Voltage (IS) | -4500 V |
| Declustering Potential (DP) | -200 V |
| Entrance Potential (EP) | -10 V |
| Collision Energy (CE) | -62 V |
| Collision Cell Exit (CXP) | -11.5 V |
| Dwell time | 20 ms |
| Injection Volume | 10 µL |

**S3 Table. Surrogate Analyte Recovery with 3 Linear Regression Models**

| Surrogate Analyte | Actual Conc. (nM) | PAR | Back-Calculated Recovery | | |
| --- | --- | --- | --- | --- | --- |
|  |  |  | Fit 1  Linear Fit | Fit 2  Linear through Zero | Fit 3  Power |
| PI (21:0\|22:6) | 0.5 | 0.03 | -3% | 73% | 98% |
|  | 1 | 0.06 | -7% | 73% | 96% |
|  | 4 | 0.29 | -45% | 88% | 111% |
|  | 10 | 0.71 | -495% | 86% | 106% |
|  | 40 | 2.67 | 112% | 81% | 95% |
|  | 100 | 6.52 | 88% | 79% | 90% |
|  | 250 | 17.76 | 89% | 86% | 96% |
|  | 500 | 37.88 | 92% | 92% | 100% |
|  | 1000 | 85.02 | 103% | 103% | 109% |
| PI (17:0\|14:1) | 0.5 | 0.04 | -2% | 43% | 73% |
|  | 1 | 0.15 | -6% | 81% | 131% |
|  | 4 | 0.59 | -33% | 80% | 117% |
|  | 10 | 1.34 | -198% | 73% | 101% |
|  | 40 | 5.69 | 115% | 77% | 98% |
|  | 100 | 14.17 | 87% | 77% | 92% |
|  | 250 | 39.20 | 88% | 85% | 96% |
|  | 500 | 82.78 | 91% | 90% | 97% |
|  | 1000 | 191.12 | 103% | 104% | 107% |

**Linearity of endogenous PI species from plasma dilution**

For each PI lipid monitored, the fold-change between the PAR from two samples are compared against the nominal fold-change. The result demonstrates the accuracy of fold-change calculation. For example, if PAR S1=A, PAR S2=B, S1 and S2 are 8x dilution and 2x dilution samples, then A/B is compared against 0.25, or $({\frac{1}{8})}/{(\frac{1}{2}}$). Similarly, B/A is compared against 4. The result is calculated for each pair of samples of the same lipid species. S2A and S2B Fig summarized the data from the evaluation of fold-change calculation accuracy of each sample compared to each other sample (containing both A/B and B/A calculation described above).

**
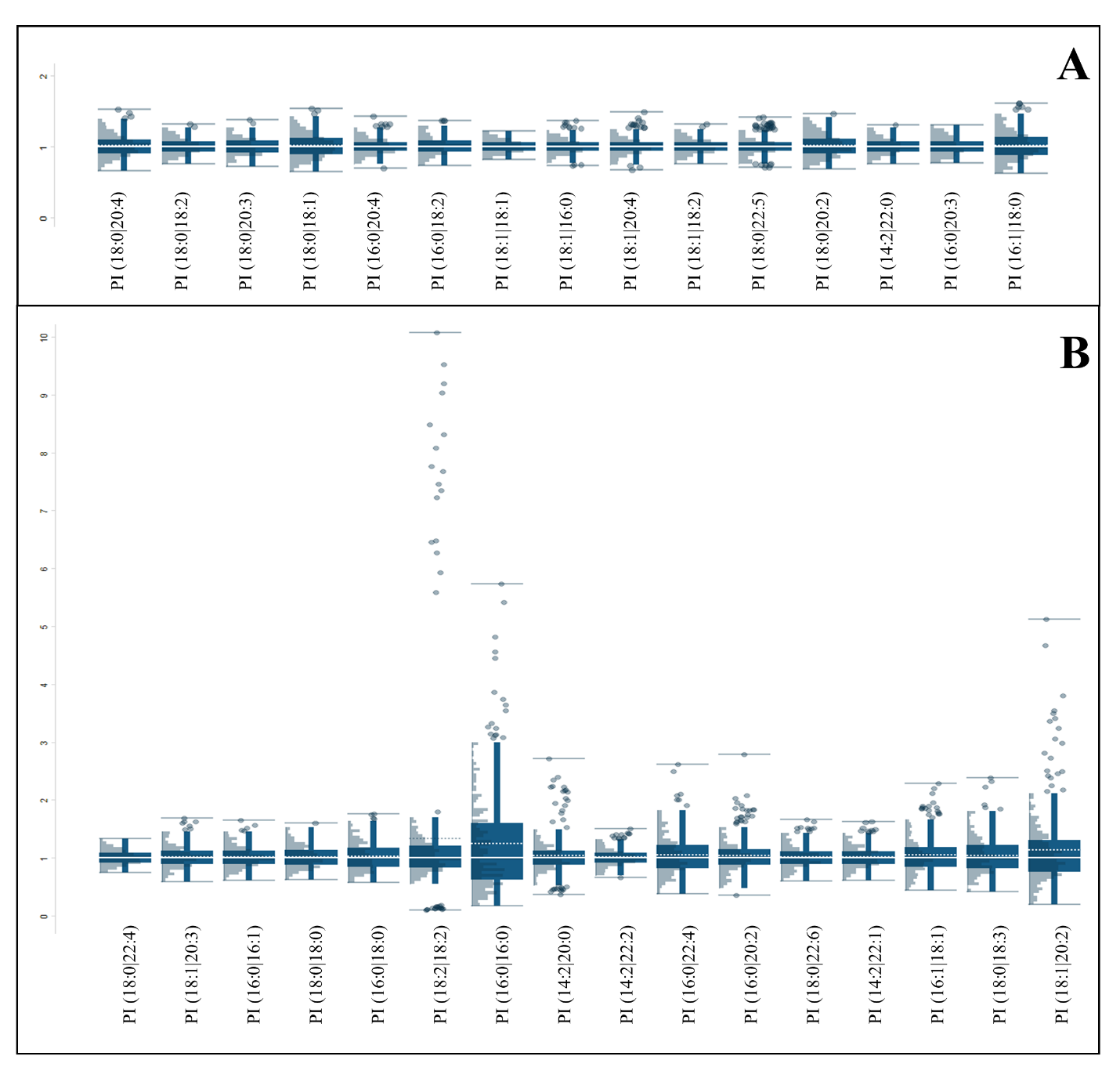
**

**S3 Fig. Linearity evaluation with parallel dilution series (1x, 2x, 3x, 4x, 6x, 8x) of endogenous human plasma PIs**. A) and B) the distribution of the fold change recovery**.**

S4 Table. Summary Statistics for Fold Change Recovery for 31 PI Species.

| Component Index | Component Name | P10^1^ | P90^1^ | L95^2^ | U95^2^ |
| --- | --- | --- | --- | --- | --- |
| 1 | PI (18:0\|20:4) | 0.83 | 1.21 | 0.99 | 1.03 |
| 2 | PI (18:0\|18:2) | 0.87 | 1.16 | 0.99 | 1.02 |
| 3 | PI (18:0\|20:3) | 0.88 | 1.14 | 0.99 | 1.02 |
| 4 | PI (18:0\|18:1) | 0.81 | 1.24 | 1.00 | 1.03 |
| 5 | PI (16:0\|20:4) | 0.88 | 1.14 | 0.99 | 1.02 |
| 6 | PI (16:0\|18:2) | 0.85 | 1.18 | 0.99 | 1.02 |
| 7 | PI (18:1\|18:1) | 0.9 | 1.11 | 0.99 | 1.01 |
| 8 | PI (18:1\|16:0) | 0.88 | 1.13 | 0.99 | 1.02 |
| 9 | PI (18:1\|20:4) | 0.87 | 1.15 | 0.99 | 1.02 |
| 10 | PI (18:1\|18:2) | 0.88 | 1.13 | 0.99 | 1.02 |
| 11 | PI (18:0\|22:5) | 0.84 | 1.19 | 0.99 | 1.02 |
| 12 | PI (18:0\|20:2) | 0.81 | 1.23 | 1.00 | 1.03 |
| 13 | PI (14:2\|22:0) | 0.88 | 1.14 | 0.99 | 1.02 |
| 14 | PI (16:0\|20:3) | 0.85 | 1.17 | 0.99 | 1.02 |
| 15 | PI (16:1\|18:0) | 0.78 | 1.29 | 1.00 | 1.04 |
| 16 | PI (18:0\|22:4) | 0.85 | 1.17 | 0.99 | 1.02 |
| 17 | PI (18:1\|20:3) | 0.81 | 1.24 | 1.00 | 1.04 |
| 18 | PI (16:0\|16:1) | 0.8 | 1.26 | 1.00 | 1.03 |
| 19 | PI (18:0\|18:0) | 0.8 | 1.26 | 1.00 | 1.04 |
| 20 | PI (16:0\|18:0) | 0.74 | 1.36 | 1.00 | 1.05 |
| 21 | PI (18:2\|18:2) | 0.68 | 1.47 | 1.16 | 1.50 |
| 22 | PI (16:0\|16:0) | 0.42 | 2.41 | 1.15 | 1.34 |
| 23 | PI (14:2\|20:0) | 0.75 | 1.33 | 1.01 | 1.08 |
| 24 | PI (14:2\|22:2) | 0.82 | 1.21 | 0.99 | 1.03 |
| 25 | PI (16:0\|22:4) | 0.65 | 1.53 | 1.01 | 1.09 |
| 26 | PI (16:0\|20:2) | 0.70 | 1.43 | 1.01 | 1.07 |
| 27 | PI (18:0\|22:6) | 0.77 | 1.29 | 1.00 | 1.04 |
| 28 | PI (14:2\|22:1) | 0.81 | 1.23 | 1.00 | 1.03 |
| 29 | PI (16:1\|18:1) | 0.66 | 1.51 | 1.01 | 1.08 |
| 30 | PI (18:0\|18:3) | 0.69 | 1.45 | 1.01 | 1.08 |
| 31 | PI (18:1\|20:2) | 0.58 | 1.71 | 1.06 | 1.20 |

1. P10 and P90 are the 10^th^ and 90^th^ percentile, respectively.

2. The L95 is the lower endpoint of the confidence interval, the U95 is the upper endpoint of the confidence interval. Confidence intervals are calculated as $\bar{x}\pm\frac{1.959964\times s}{\sqrt{n}}$; where $\bar{x}$ is the average value of the group, s is the sample standard deviation, n is the number of values in the group and the static t-value of 1.959964 was used as the dataset contained more than 40 values per group.

**Stability of the plasma samples and the extracted samples**

The evaluation of the short-term stability include: 1) benchtop stability, with samples placed on ice for 2 hours or in a 2-8⁰ C refrigerator for overnight; and 2) freeze-thaw stability at two or three cycles. To assess plasma sample stability we compared the peak area ratio of treated sample to freshly prepared sample at two concentrations, LQC and HQC. For each freeze-thaw cycle, plasma was frozen at ≤-60 ⁰C for at least 12 hours before completely thawed under 2-8 ⁰C. The data is summarized in S1 Fig. In general, the majority of lipid species at HQC level were stable for all four conditions, except for the PI (16:0|16:0), which is a lower abundance species. For LQC samples, although most of the higher signal response species were stable with a recovery between 70-130%, a general decrease was observed across the species. The lower abundance species appear to be much more susceptible to loss under stressed conditions. Among the 31 species, PI (18:0|18:3) and PI (18:1|20:2) did not meet the acceptance criteria for all conditions. This suggests that the although the PI in human plasma samples are generally stable, they need to be stored without dilution and the time exposed to bench top operations should be minimized. When samples are retested after multiple freeze-thaws, the less stable (lower abundance) PI species should not be included in quantitative assessments.


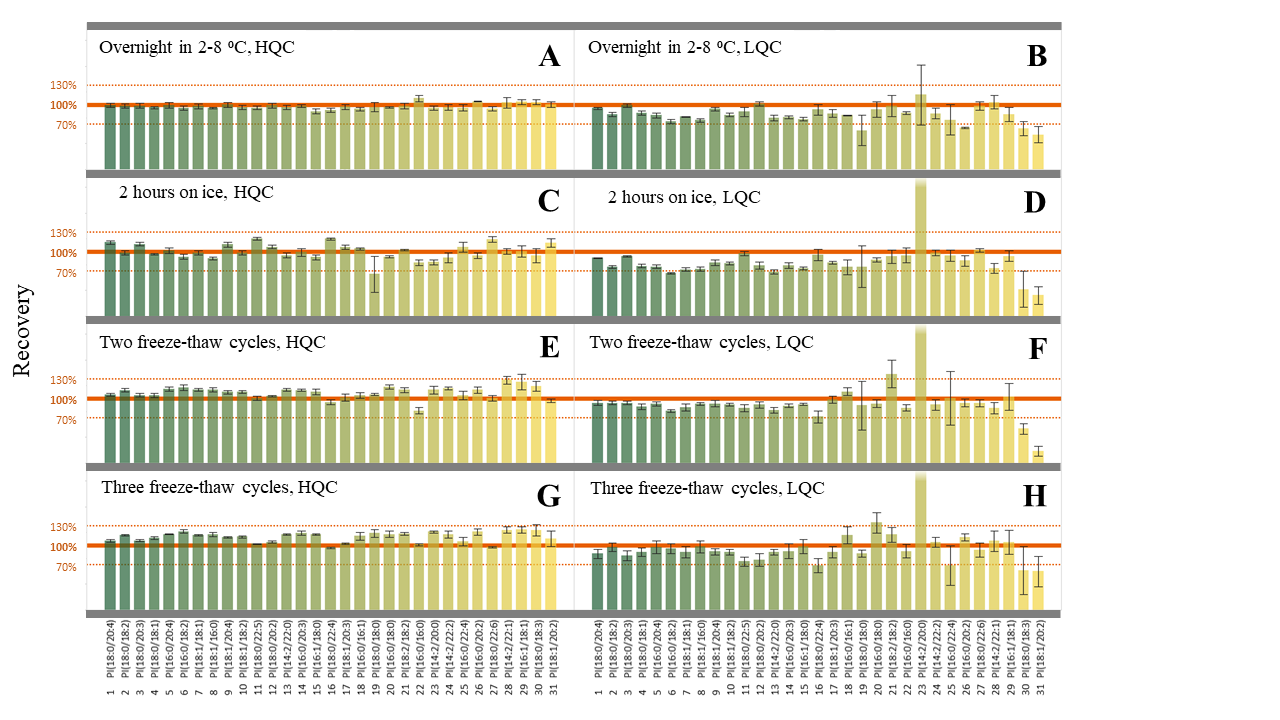


**S4 Fig. Short-term stability evaluation. Recovery for LQC (B, D, E, F) and HQC (A, C, E, G) under different stressed conditions, including overnight storage in 2-8 ⁰C (A, B), 2 hr storage on ice (C, D), freeze-thaw for two cycles (E, F) and three cycles (G, F).**

The run length evaluation is performed by comparing the PAR of the HQC and LQC throughout the run. The run length of the assay is proven to be stable for 108 injections. The reinjection stability for the extracted samples was also evaluated by comparing the PAR of the reinjected sample with the PAR of the same sample at first injection. The HQC/LQC ratio was calculated for each species in the original injection and the repeated injection. From the results, although the top 15 PI species showed relatively comparable pass/fail rate for the LQC and HQC, the lower abundance species appeared to fall out of the 70%-130% acceptance criteria more frequently compared to the original injection. Thus, it is recommended the samples be injected immediately after extraction and the number of samples prepared in one batch to be limited to below 96, including the qualities control samples.

**S5 Table. Baseline PI in healthy individuals. Reference range PAR of the monitored PI species in 49 healthy individuals**

| PI species | Average PAR | Median PAR | Standard Deviation | CV% | Reference  Range ^a^ |
| --- | --- | --- | --- | --- | --- |
| PI (18:0\|20:4) | 15.72 | 13.52 | 9.00 | 57% | 0-33.72 |
| PI (18:0\|18:2) | 5.99 | 4.92 | 3.90 | 65% | 0-13.79 |
| PI (18:0\|20:3) | 3.30 | 2.78 | 2.35 | 71% | 0-7.99 |
| PI (18:0\|18:1) | 2.46 | 2.03 | 1.44 | 59% | 0-5.34 |
| PI (16:0\|20:4) | 1.93 | 1.64 | 1.41 | 73% | 0-4.75 |
| PI (16:0\|18:2) | 2.44 | 2.26 | 1.65 | 68% | 0-5.74 |
| PI (18:1\|18:1) | 1.65 | 1.36 | 0.96 | 58% | 0-3.56 |
| PI (18:1\|16:0) | 2.05 | 1.74 | 1.31 | 64% | 0-4.68 |
| PI (18:1\|20:4) | 0.73 | 0.60 | 0.49 | 68% | 0-1.72 |
| PI (18:1\|18:2) | 0.95 | 0.85 | 0.51 | 53% | 0-1.96 |
| PI (18:0\|22:5) | 0.40 | 0.30 | 0.45 | 113% | 0-1.30 |
| PI (18:0\|20:2) | 0.30 | 0.26 | 0.20 | 68% | 0-0.71 |
| PI (14:2\|22:0) | 0.49 | 0.43 | 0.32 | 65% | 0-1.13 |
| PI (16:0\|20:3) | 0.50 | 0.41 | 0.39 | 79% | 0-1.28 |
| PI (16:1\|18:0) | 0.54 | 0.34 | 0.74 | 138% | 0-2.02 |
| PI (18:0\|22:4) | 0.19 | 0.15 | 0.16 | 86% | 0-0.50 |
| PI (18:1\|20:3) | 0.15 | 0.13 | 0.11 | 74% | 0-0.38 |
| PI (16:0\|16:1) | 0.37 | 0.16 | 0.49 | 133% | 0-1.34 |
| PI (18:0\|18:0) | 0.15 | 0.13 | 0.13 | 88% | 0-0.42 |
| PI (16:0\|18:0) | 0.14 | 0.13 | 0.10 | 69% | 0-0.34 |
| PI (18:2\|18:2) | 0.10 | 0.08 | 0.08 | 81% | 0-0.26 |
| PI (16:0\|16:0) | 0.21 | 0.17 | 0.20 | 94% | 0-0.62 |
| PI (14:2\|20:0) | 0.14 | 0.15 | 0.11 | 78% | 0-0.37 |
| PI (14:2\|22:2) | 0.14 | 0.12 | 0.10 | 73% | 0-0.35 |
| PI (16:0\|22:4) | 0.08 | 0.07 | 0.09 | 103% | 0-0.26 |
| PI (16:0\|20:2) | 0.09 | 0.08 | 0.06 | 71% | 0-0.21 |
| PI (18:0\|22:6) | 0.37 | 0.30 | 0.26 | 70% | 0-0.88 |
| PI (14:2\|22:1) | 0.11 | 0.10 | 0.07 | 66% | 0-0.26 |
| PI (16:1\|18:1) | 0.10 | 0.08 | 0.11 | 111% | 0-0.33 |
| PI (18:0\|18:3) | 0.07 | 0.06 | 0.09 | 114% | 0-0.25 |
| PI (18:1\|20:2) | 0.02 | 0.01 | 0.02 | 106% | 0-0.07 |

^a^ Reference range was calculated as mean ± 2*standard deviation.

**S6 Table. Demographic Information of 49 Healthy Individuals**

| Female/Male (n) | 25/24 |
| --- | --- |
| Age (y) | 18-61, Median=43 |
| BMI | 20-52, Median=27.5 |
| Total Cholesterol (mg/dL) | 98-274, Median=158 |
| HDL (mg/dL) | 18-85, Median= 40 |
| LDL (mg/dL) | 6.6-149.6, Median=70.8 |

**S7 Table. Demographic and General Lipid Information for the 49 Healthy Individual Donors**

| **BIOREC ID#** | **Lot Number** | **Blood Type** | **Gender** | **Race** | **Age** | **BMI** | **Total Cholesterol** | **Triglycerides** | **HDL** | **LDL** | **Lipemia Level** |
| --- | --- | --- | --- | --- | --- | --- | --- | --- | --- | --- | --- |
| HML1587 | BRH1481411 | O | F | HB | 30 | 29 | 145 | 254 | 64 | 30.2 | Low |
| HML1190 | BRH1481412 | B | F | O | 29 | 22 | 137 | 152 | 45 | 61.6 | Medium |
| HML2119 | BRH1481413 | O+ | F | H | 19 | 24 | 159 | 179 | 42 | 81.2 | Low |
| HML1673 | BRH1481414 | O- | F | C | 19 | 26 | 134 | 188 | 41 | 55.4 | Medium |
| HML1096 | BRH1481415 | O+ | F | B | 20 | 43 | 158 | 133 | 36 | 95.4 | Medium |
| HML1916 | BRH1481416 | A+ | F | B | 18 | 26 | 177 | 241 | 33 | 95.8 | Medium |
| HML1030 | BRH1481417 | O+ | F | B | 27 | 28 | 113 | 120 | 29 | 60 | Medium |
| HML0933 | BRH1481418 | A | F | B | 26 | 38 | 222 | 232 | 26 | 149.6 | NA |
| HML2114 | BRH1481419 | A+ | F | H | 26 | 52 | 122 | 623 | 20 | NA | High |
| HML1206 | BRH1481420 | A | M | B | 29 | 27 | 137 | 85 | 85 | 35 | Low |
| HML1334 | BRH1481421 | A | M | B | 28 | 49 | 127 | 227 | 73 | 8.6 | Medium |
| HML1226 | BRH1481422 | O | M | HB | 24 | 29 | 164 | 116 | 48 | 92.8 | Medium |
| HML1935 | BRH1481423 | A+ | M | B | 21 | 25 | 98 | 176 | 46 | 16.8 | Low |
| HML0610 | BRH1481424 | AB | M | H | 28 | NA | 139 | 136 | 41 | 70.8 | Medium |
| HML1850 | BRH1481425 | O+ | M | A | 27 | 29 | 121 | 94 | 35 | 67.2 | Medium |
| HML1938 | BRH1481426 | A+ | M | H | 28 | 24 | 193 | 268 | 29 | 110.4 | Low |
| HML1557 | BRH1481427 | O+ | M | B | 27 | 46 | 175 | 479 | 26 | 53.2 | Medium |
| HML2110 | BRH1481428 | O+ | F | H | 44 | 25 | 152 | 214 | 64 | 45.2 | Medium |
| HML1734 | BRH1481429 | B | F | B | 40 | 34 | 162 | 221 | 47 | 70.8 | Medium |
| HML1689 | BRH1481430 | O+ | F | H | 44 | 25 | 141 | 163 | 42 | 66.4 | Low |
| HML1864 | BRH1481431 | O | F | B | 38 | 36 | 170 | 203 | 40 | 89.4 | Medium |
| HML0777 | BRH1481432 | O | F | HC | 33 | 30 | 217 | 194 | 34 | 144.2 | Low |
| HML1690 | BRH1481433 | B+ | F | H | 41 | 26 | 101 | 153 | 30 | 40.4 | Low |
| HML1949 | BRH1481434 | A+ | F | O | 40 | 44 | 144 | 212 | 22 | 79.6 | Low |
| HML1506 | BRH1481435 | O+ | F | Other | 36 | 34 | 164 | 475 | 21 | 48 | Medium |
| HML0929 | BRH1481436 | A+ | M | B | 47 | 23 | 168 | 169 | 72 | 62.2 | NA |
| HML0737 | BRH1481437 | A+ | M | B | 47 | NA | 182 | 211 | 58 | 81.8 | NA |
| HML1971 | BRH1481438 | B+ | M | C | 40 | 26 | 188 | 166 | 46 | 108.8 | Low |
| HML2000 | BRH1481439 | A+ | M | H | 44 | 32 | 274 | 511 | 43 | 128.8 | Low |
| HML0449 | BRH1481440 | O | M | B | 48 | 26 | 129 | 156 | 38 | 59.8 | Medium |
| HML0893 | BRH1481441 | O | M | HC | 48 | 34 | 202 | 478 | 30 | 76.4 | Medium |
| HML1069 | BRH1481442 | A+ | M | C | 45 | 43 | 145 | 277 | 21 | 68.6 | Medium |
| HML2007 | BRH1481443 | A+ | M | H | 43 | 28 | 213 | 808 | 21 | 30.4 | High |
| HML1844 | BRH1481444 | A+ | F | B | 61 | 20 | 178 | 148 | 70 | 78.4 | Low |
| HML1654 | BRH1481445 | A+ | F | B | 52 | 28 | 223 | 248 | 48 | 125.4 | Low |
| HML1184 | BRH1481446 | O+ | F | B | 59 | 29 | 139 | 236 | 45 | 46.8 | Medium |
| HML1714 | BRH1481447 | O+ | F | H | 53 | 27 | 206 | 296 | 37 | 109.8 | Low |
| HML0425 | BRH1481448 | A | F | B | 51 | 35 | 172 | 189 | 34 | 100.2 | Medium |
| HML2112 | BRH1481449 | O+ | F | H | 53 | 27 | 211 | 365 | 31 | 107 | Low |
| HML1713 | BRH1481450 | O+ | F | H | 55 | 21 | 226 | 843 | 28 | 29.4 | Low |
| HML1945 | BRH1481451 | O+ | F | H | 56 | 25 | 158 | 519 | 21 | 33.2 | High |
| HML1602 | BRH1481452 | A+ | M | H | 56 | 33 | 180 | 54 | 69 | 100.2 | Low |
| HML1189 | BRH1481453 | A | M | B | 61 | 24 | 157 | 118 | 53 | 80.4 | Low |
| HML1846 | BRH1481454 | B+ | M | B | 53 | 29 | 203 | 222 | 48 | 110.6 | Low |
| HML1849 | BRH1481455 | O+ | M | H | 55 | 22 | 153 | 144 | 46 | 78.2 | Low |
| HML0795 | BRH1481456 | A- | M | C | 57 | NA | 149 | 282 | 42 | 50.6 | Medium |
| HML1016 | BRH1481457 | O+ | M | H | 53 | 27 | 156 | 226 | 36 | 74.8 | Low |
| HML2003 | BRH1481458 | O+ | M | B | 52 | 27 | 127 | 260 | 29 | 46 | Medium |
| HML1563 | BRH1481459 | B | M | C | 55 | 24 | 131 | 532 | 18 | 6.6 | Medium |

All information in S6 and S7 Tables was provided by BioIVT.

**
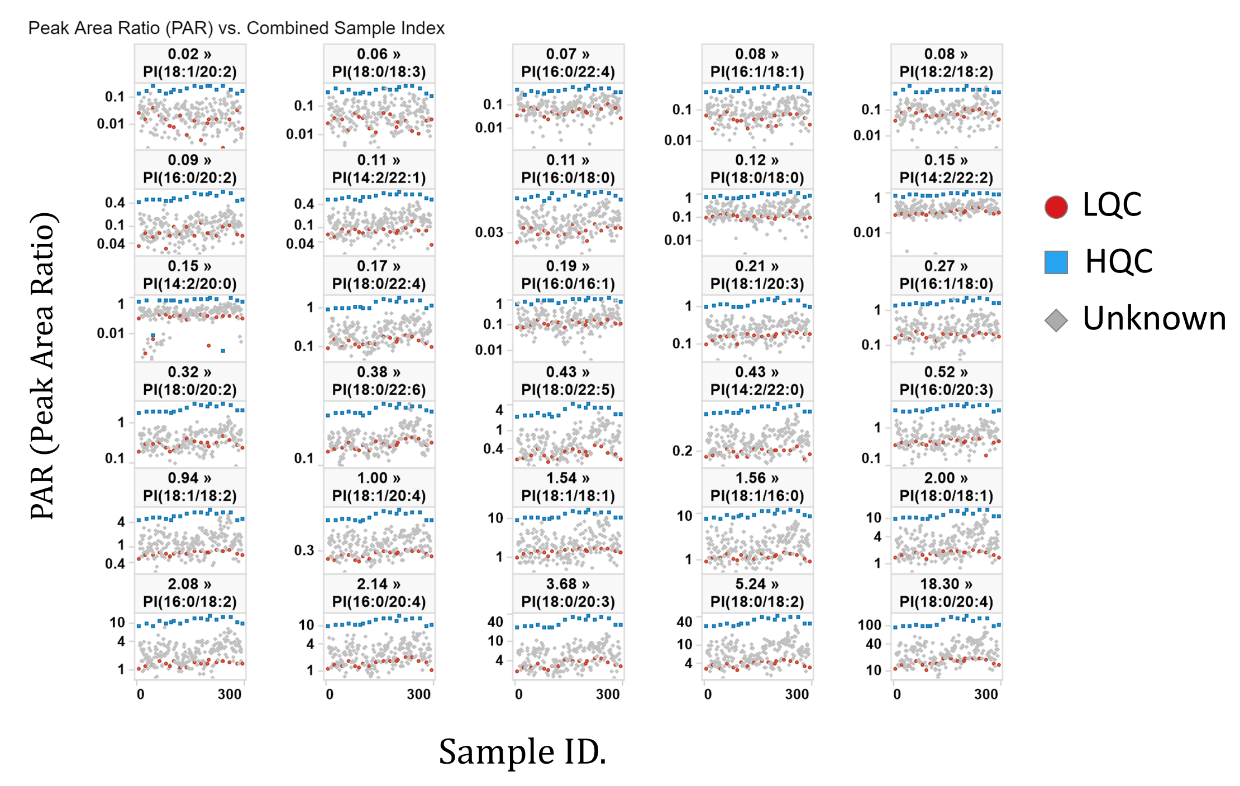
**

**S5A Fig. Level of PI quality controls (LQC and HQC) relative to study samples for SAD study.**

**
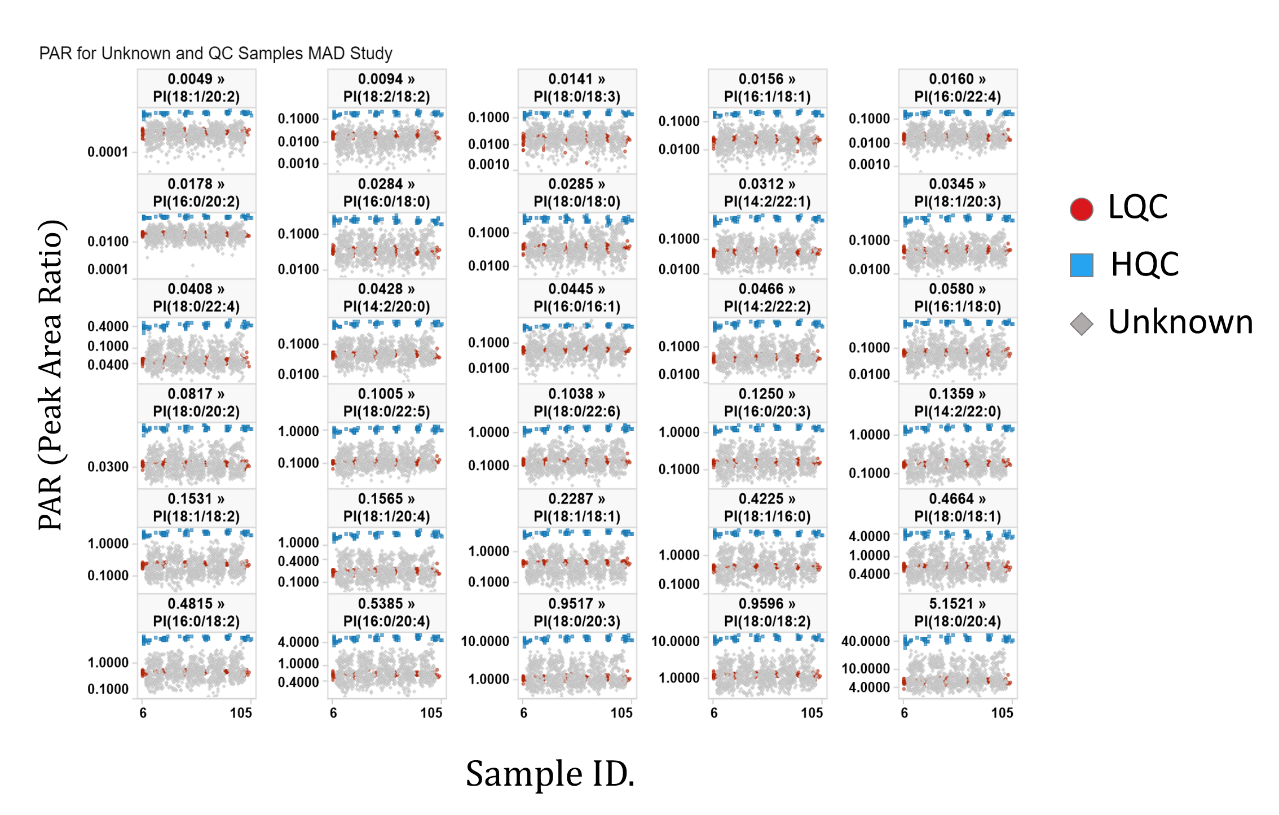
**

**S5B Fig. Level of PI quality controls (LQC and HQC) relative to study samples for MAD study.**

**
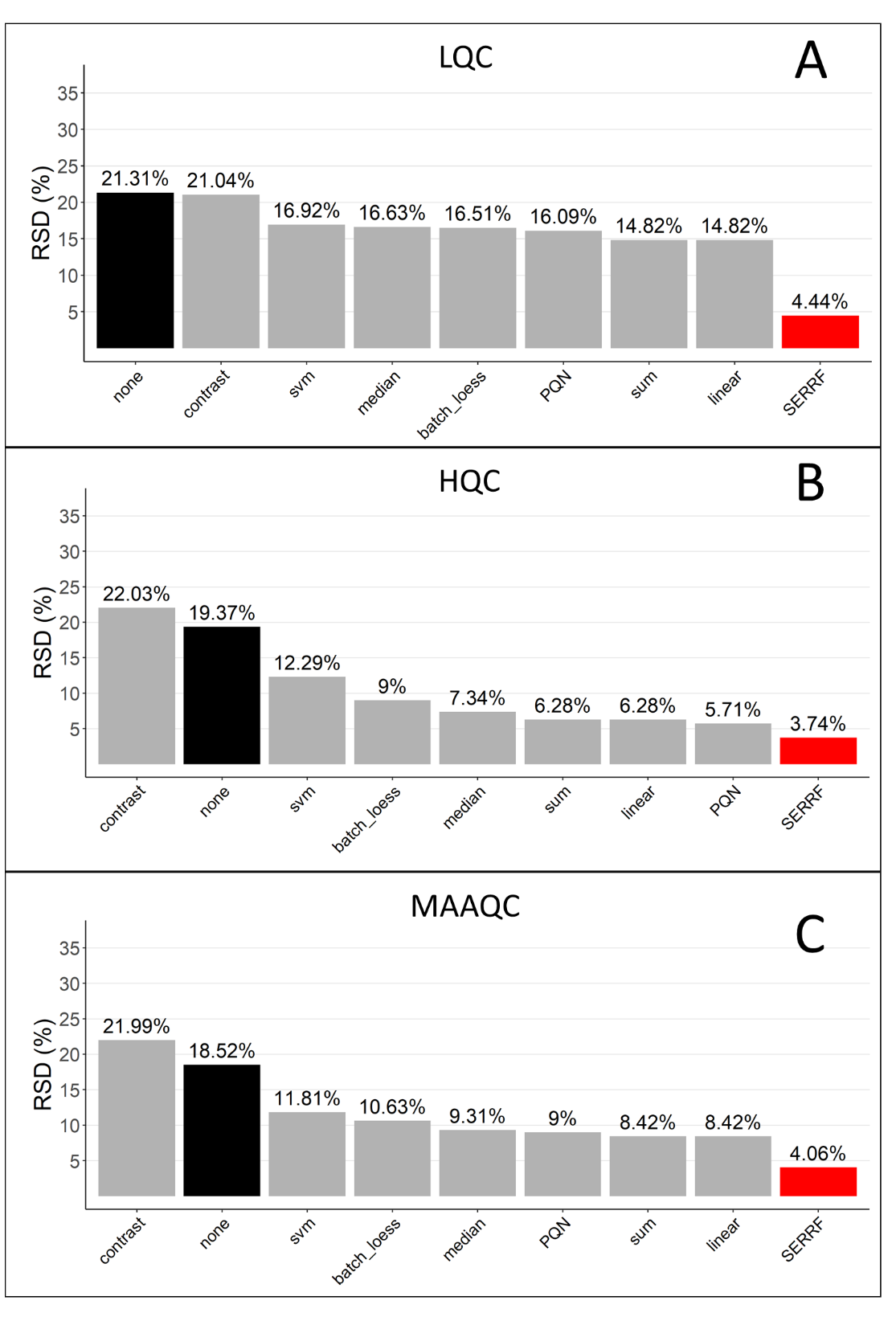
**

**S6 Fig. Combined RSD (%) for all PI species for several normalization methods using LQC (panel A), HQC (panel B) or MAAQC (mean-adjusted average QC, panel C).** SERRF outperformed all evaluated normalization methods for either QC**.**

**
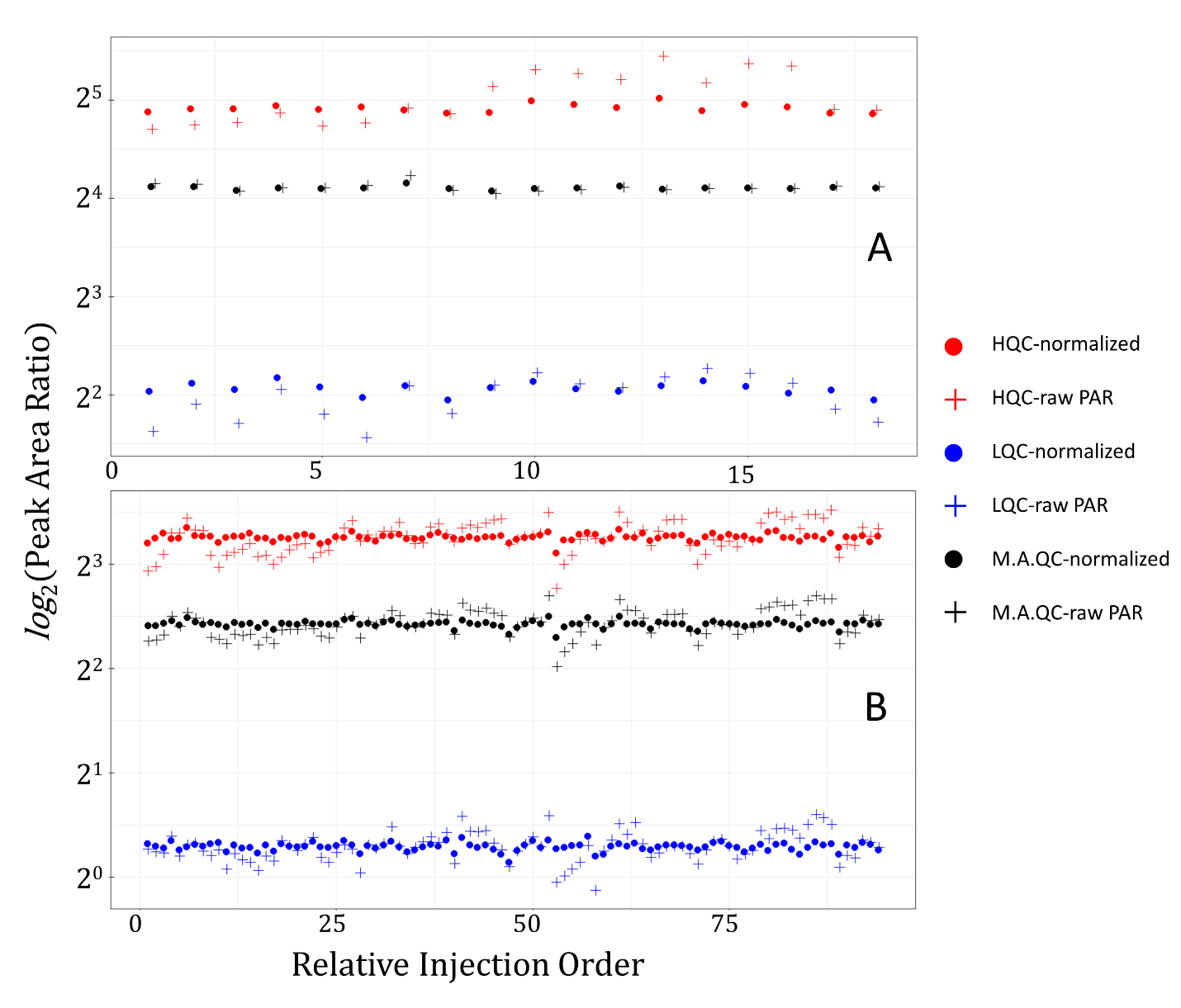
 S7 Fig. Level of quality control samples (LQC, HQC and MAAQC) for PI (18:0|18:2) before and after normalization for SAD study (panel A) and MAD study (panel B).** The normalization was evident comparing the before normalization (+) and after normalization (●) with SERRF.

**
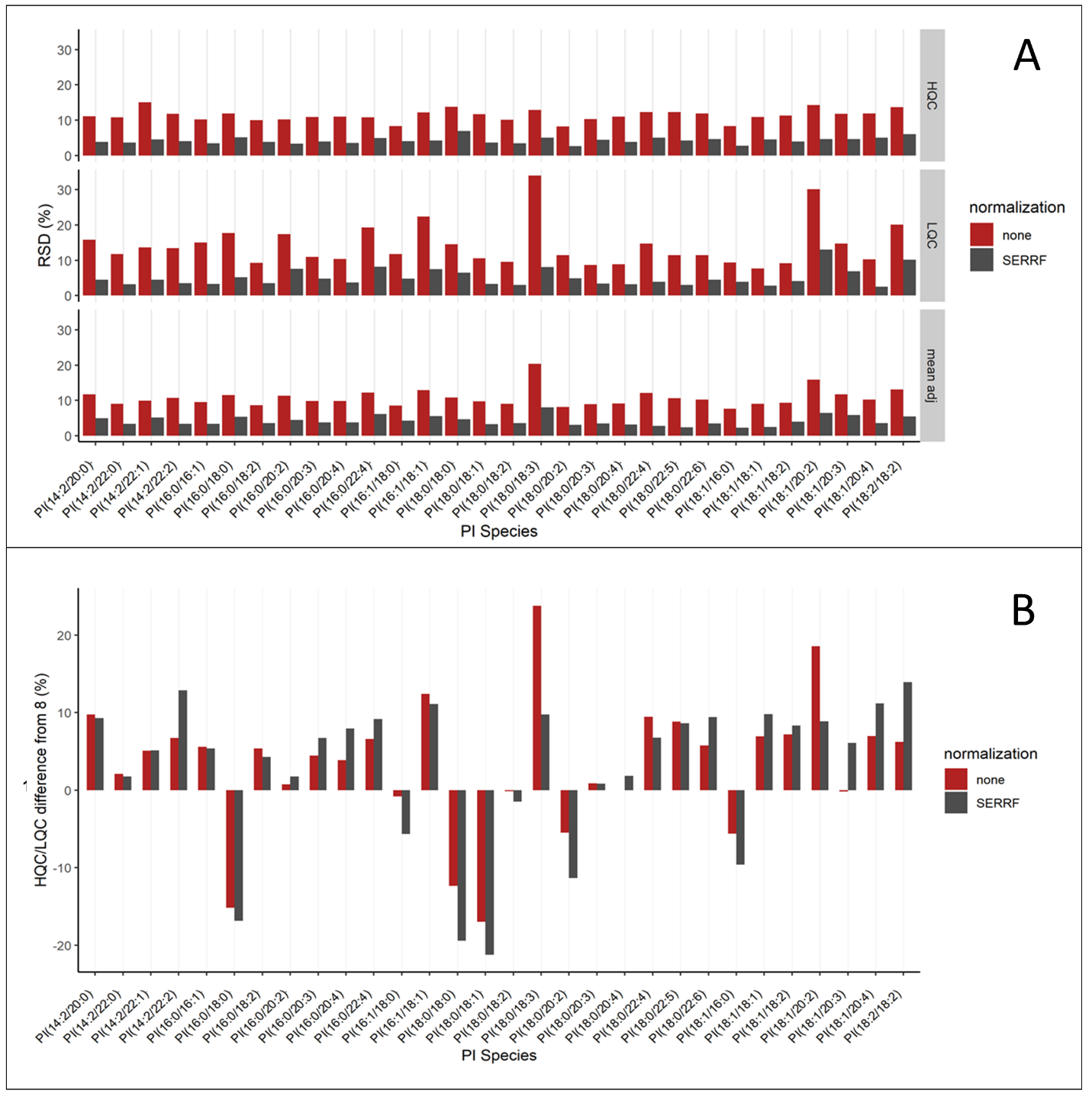
 S8 Fig. A) RSD (%) for each PI species monitored with and without SERRF demonstrating reduction in RSD for each PI species with SERRF**. B) HQC/LQC ratio difference from nominal (8). Dilutional linearity was maintained after normalization with SERRF across PI species.

**
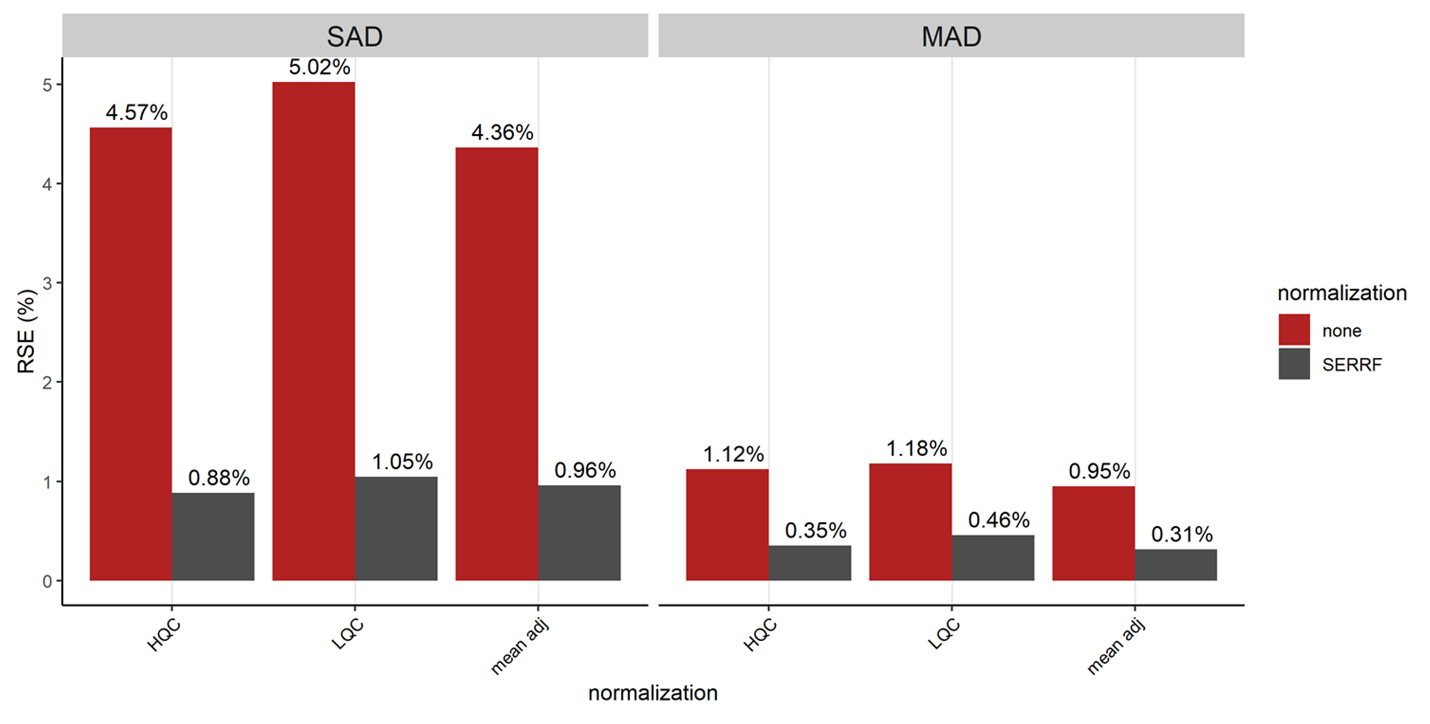
 S9 Fig. Comparison of the RSE (%) with and without SERRF for SAD and MAD study.** Automation used for MAD study likely decreased the raw data variability.

**
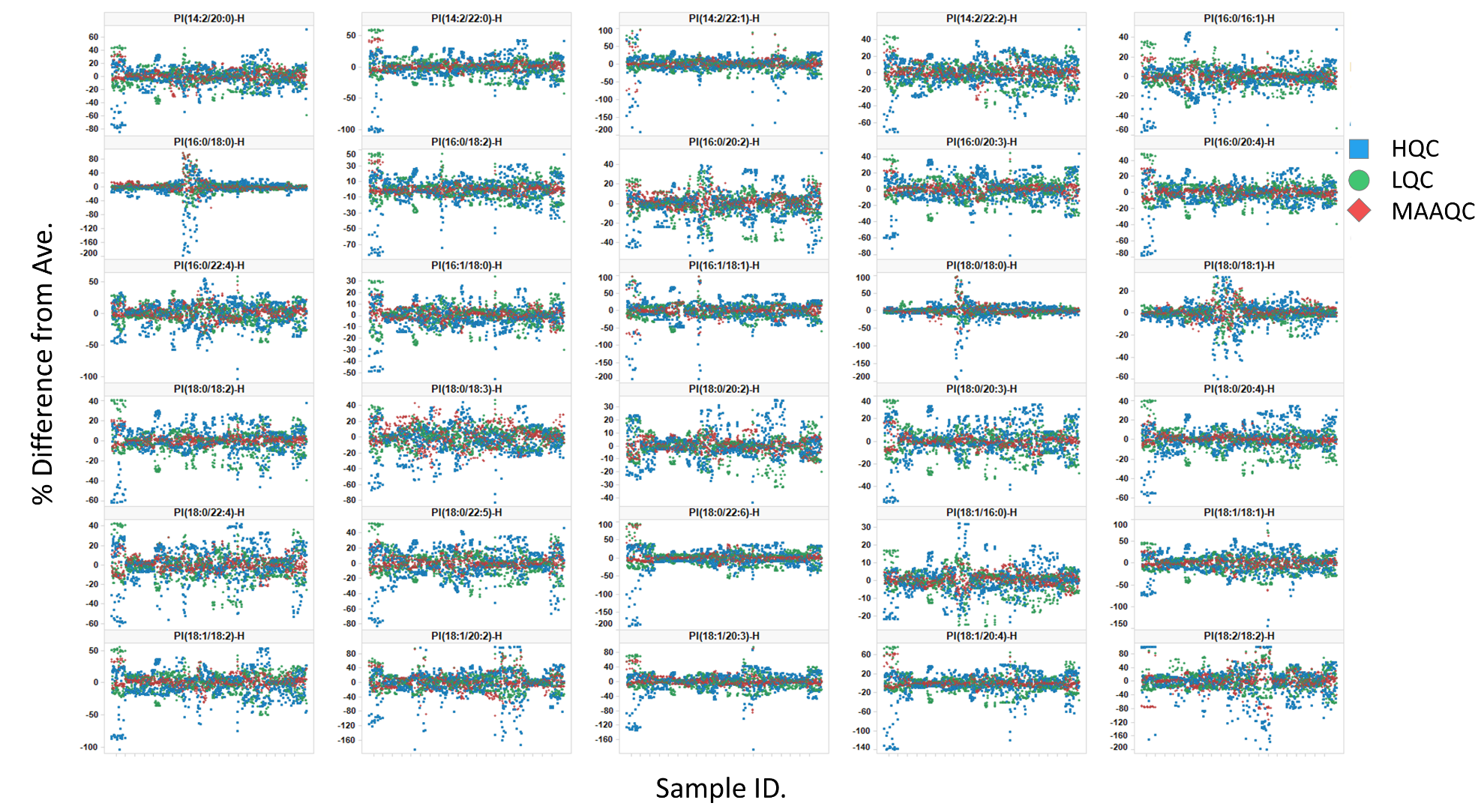
**

**S10 Fig. % Difference of normalized value using LQC, HQC and MAAQC from the average of these three values for MAD study samples**. Each panel represents one endogenous PI species.


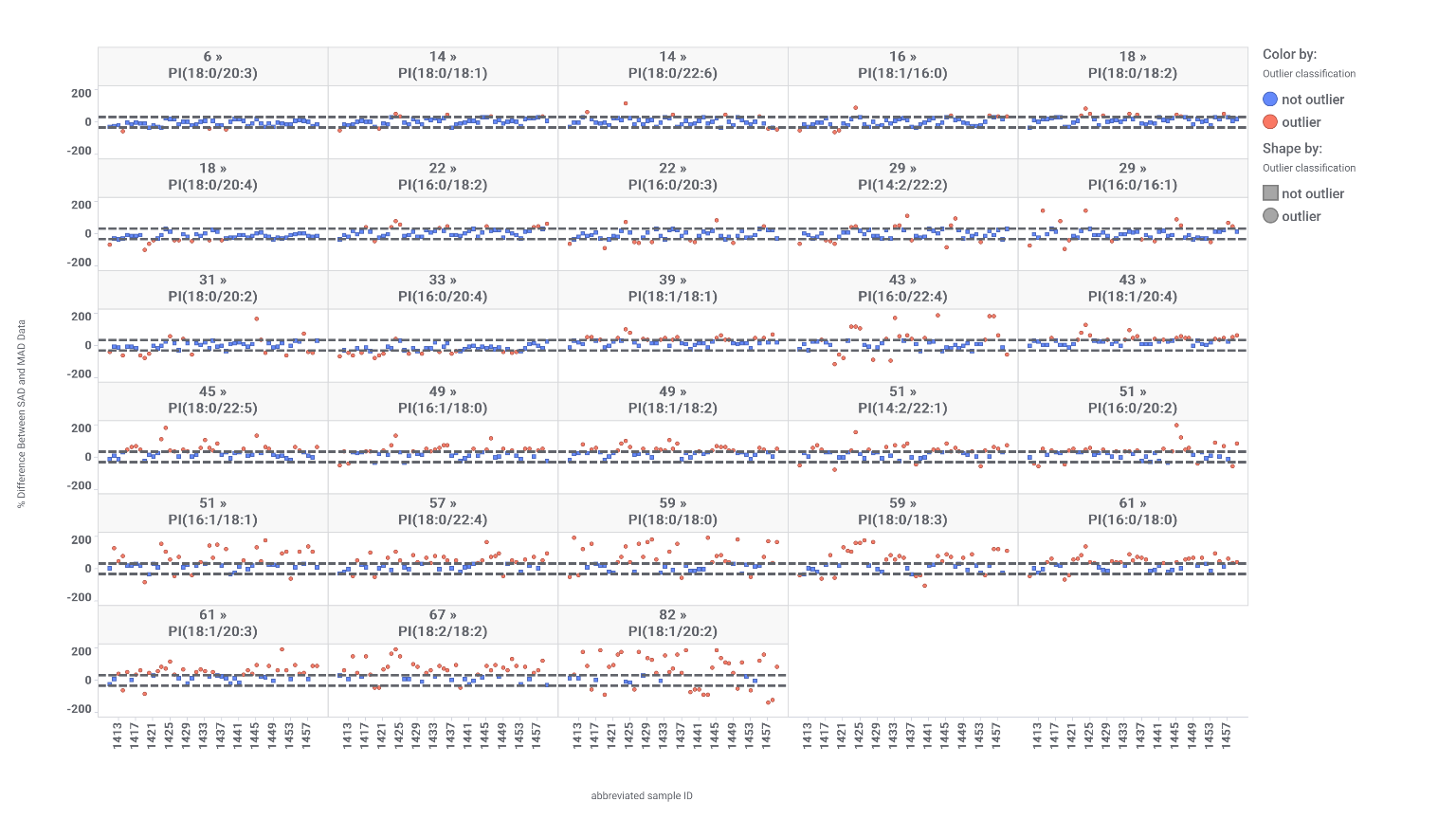


**S11 Fig.** **Percent Difference of each PI species for 49 Healthy individual samples analyzed along with the two clinical studies.** Outliers were samples with % Difference >±35. Numbers above PI species name indicate % outlier for each PI species.


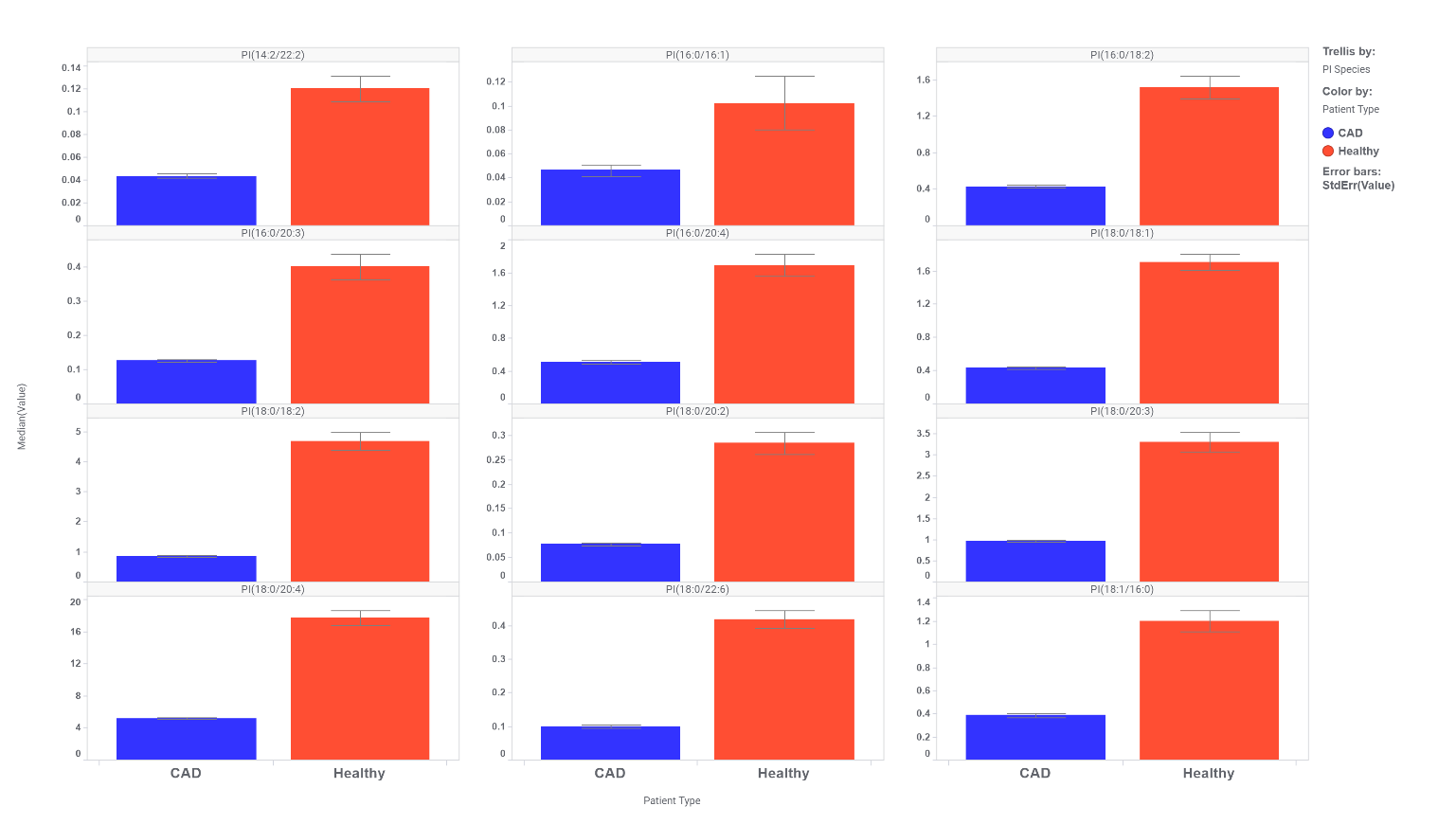


**S12 Fig. Comparison of healthy volunteer and CAD patient PI PAR values from the two clinical trials on day 1 (before any treatment).** Values are presented as median for each group per PI species. Error bars are std. error (n=32 for healthy, n= 126 for CAD). The difference was determined to be statistically significant by 2-tailed heteroscedastic t-test, p<0.0005 for all 12 PI species.
